# The role of ubiquitin system in ageing-related diseases and potential therapeutic opportunities

**DOI:** 10.1186/s43556-026-00494-5

**Published:** 2026-06-26

**Authors:** Xiaoya Ren, Xin Zhou, Zhiqiang Ma, Dong Liu

**Affiliations:** 1 State Key Laboratory of Cardiovascular Disease, Fuwai Hospital, National Center for Cardiovascular Diseases, Chinese Academy of Medical Sciences, Peking Union Medical College, 167 Beilishi Road, Beijing, 100037 China; 2 Department of Medical Oncology, Senior Department of Oncology, Chinese PLA General Hospital, The Fifth Medical Center, 28 Fuxing Road, Beijing, 100853 China; 3 Graduate School of Chinese Academy of Medical Science and Peking Union Medical College, 9 Dongdan Santiao Alley, Beijing, 100001 China

**Keywords:** Ubiquitin proteasome system, DUBs, Ageing-related diseases, Cancers

## Abstract

Ubiquitination functions significantly in ageing, contributing to its influences on ageing-related diseases. Being one of the important posttranslational modifications, ubiquitination can make a difference to the stabilization of diverse proteins, which can be reversed by deubiquitinases (DUBs). The mechanisms of ageing such as loss of proteostasis, mitochondrial dysfunction are gradually illustrated and researchers’ attention on ageing-related diseases including cancers is increasing. The association between ubiquitin proteasome system (UPS) and DUBs with cellular ageing and ageing-related diseases are gradually demonstrated while not comprehensively reviewed. In this review, we illustrate the relationship between ubiquitin system with ageing and provide a further introduction of roles of ubiquitin system in ageing-related diseases. To begin with, the fundamentals of ubiquitin system including the ligases, deubiquitinases and processes of ubiquitination/deubiquitination are given. Then, association between ubiquitin and cellular ageing is further discussed. Besides, we concentrate on the detailed roles of deubiquitination in ageing-related diseases, potential therapeutic targets of the ubiquitin system in ageing-related diseases and the relevant clinical trials so that we can have a comprehensive perspective of the clinical potentials of ubiquitin system in ageing-related diseases. In summary, the information concluded here may be assistant for further investigation in ageing-related diseases in the future.

## Introduction

Achieved by a three-enzyme cascade containing E1 Ub-activating enzyme, E2 Ub-conjugating enzyme and E3 Ub-protein ligase, ubiquitination is one of the essential post-translational modifications (PTMs), which can make a big difference to the regulation of a great number of cellular processes through its impacts on the functional status of diverse proteins [1, 2]. And as other posttranslational modifications, via ubiquitin cleaving from substrate proteins, ubiquitin chains editing and ubiquitin precursors processing, ubiquitination can be turned over by deubiquitinating enzymes (DUBs) [3]. Moreover, DUBs are classified into seven families in accordance with the distinctive sequence and domain conservation, involving ubiquitin-specific proteases (USPs), ubiquitin carboxy-terminal hydrolases (UCHs), Machado–Josephin domain-containing proteases (MJDs), ovarian tumor proteases (OTUs), motif-interacting with ubiquitin-containing novel DUB family (MINDYs), the JAB1/MPN/MOV34 metalloproteases (JAMMs) and ZUP1 [4].

With water, food, lifestyle and medical care that are of better quality, a doubled average human life expectancy has been observed over the past 200 years in most developed countries. Nevertheless, longer lifespan is not accompanied by longer disease-free lifespan [5]. Ageing, the physiological and metabolic deterioration related with almost every organ and system, is closely relevant with cellular ageing related processes and associated with varieties of disorders including cancers and cardiovascular diseases [6–8]. Recently, researchers gradually concentrate on the relationship between ubiquitin system and ageing, including underlying mechanisms and ageing-related diseases. For instance, the functions of quantities of DUBs on cancers such as the promotion of proliferation caused by ATXN3, POH1 and A20 have been reviewed [9]. And nowadays ubiquitination proteasome system and DUBs are emerging as new targets for treating ageing-related diseases. All above promote our focus on the detailed investigation of the association between ubiquitin system and ageing-related diseases.

In this review, we make a systematic conclusion concerning the association between ubiquitin system and ageing-related diseases and potential therapeutic opportunities. First of all, we give a brief introduction of the fundamentals of ubiquitination and deubiquitination, involving the ligases and deubiquitinases, process of ubiquitination and deubiquitination, ub-binding proteins and PTM crosstalk. Moreover, we illustrate the relationship between ubiquitin system and cellular ageing. Furthermore, we make a detailed illustration of the association between ubiquitin system and ageing-related diseases and we mainly concentrate on DUBs. Further potential therapeutic targets associated with ubiquitin system in ageing related diseases and relevant clinical trials are also introduced. Finally, some new related perspectives and potential directions are clarified. Taken together, referred to be as the reference of relationship between the ubiquitin system and aging-related diseases, the conclusion here may give the investigators a hand in the future.

## Fundamentals of the ubiquitin system

### Core enzymes in ubiquitin system

Ubiquitin, a highly conserved 76-amino-acid protein found in all tissues of eukaryotic, is a posttranslational modifiers that can connect with lysine residues and then results in the effects on diverse cellular functions [10]. With the assistance of E1 Ub-activating enzyme, E2 Ub-conjugating enzyme and E3 Ub-protein ligase, the protein modification caused by ubiquitin, also known as ubiquitination is achieved [2]. Reversibly, Ub-specific proteases, also called deubiquitinases, can remove ubiquitin from substrates [11].

E1 enzymes, involving the ubiquitin activating enzymes UAE and UBA6, contain three domains --- an adenylation domain composed of two ThiF-homology motifs, the catalytic cysteine domain and the C-terminal ubiquitin-fold domain. The domains possess the ability to bind ATP and the appropriate UBL, carry acyl for ubiquitin and recruit specific E2 enzymes respectively [12]. Characterized by a conserved ubiquitin conjugating domain, E2 conjugating enzymes can interact with E1 enzymes and E3 ligases mediated by conserved and partial overlapping interfaces [13]. E3 ligases are divided into two categories --- RING type E3s and the HECT-type E3s, which are featured by the RING or U-box fold catalytic domain and a homologous C-terminal catalytic domain which adopts a characteristic bilobal structure respectively. Furthermore, the former can lead to the promotion of Ub transfer from E2 to substrates directly, while the ubiquitination caused by HECT-type E3s contains an intermediate step where the ubiquitin is transferred from E2 to the HECT E3 ligase’s residue initially [14, 15].

Just as we have illustrated above, DUBs are currently drawn from seven conserved families and six of them are classified as cysteine proteases, including USPs, UCHs, OTUs, MJDs, MINDY and ZUP1 while JAMM family (also known as MPN +) consists of zinc-dependent metalloproteinases. Except for MJDs, each family of DUBs is conserved from yeast to human. Moreover, among 99 DUBs, 11 DUBs can still function crucially without residues critical for DUB activity and thus they are considered to be as pseudoenzymes, which are common in JAMM family [11, 16]. Among the seven DUB families, five structurally distinct families have been described extensively while the MINDY family and ZUP1 family are still under explored [17, 18].

### Mechanisms of Ubiquitination/Deubiquitination and ub-binding receptors

**Fig. 1:**
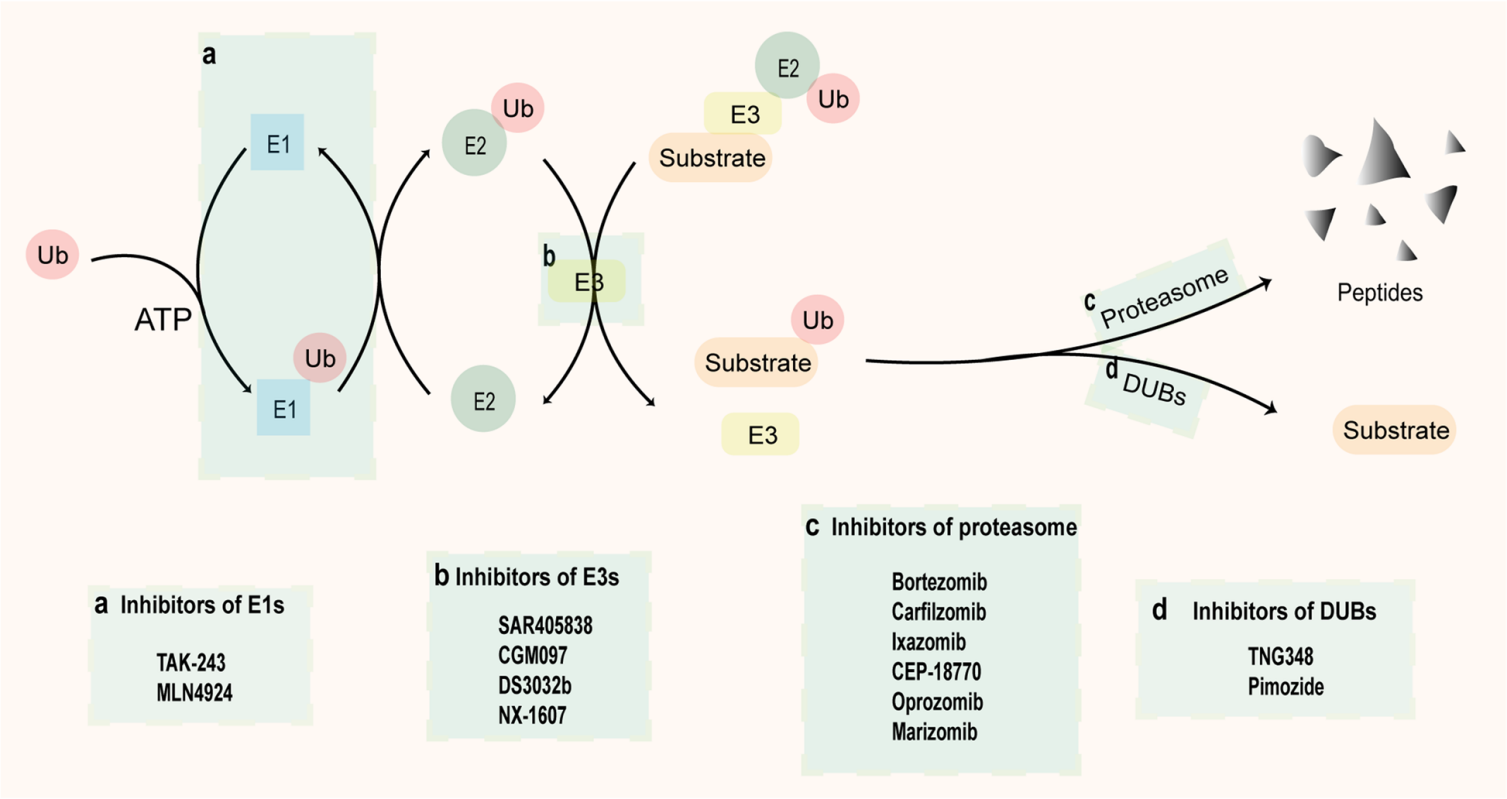
Ubiquitin system associated mechanisms and inhibitors. Ubiquitination of substrates is achieved by sequential catalyzation caused by E1, E2 and E3 contributing to ubiquitin activation, ubiquitin conjugation and ubiquitin ligation respectively. Ubiquitination of substrates results in the proteasomal degradation of themselves while ubiquitination can be reversed by DUBs. Besides, in accordance with the association between ubiquitin system and ageing-related disease, ubiquitin system related inhibitors including E1 ligase inhibitors, E3 ligase inhibitors and DUB inhibitors are gradually applicated in clinical trials

To begin with, we will introduce the process of ubiquitination. In an adenosine triphosphate-dependent manner, E1 contributes to the activation of Ub and mediates its transfer to E2, and then a thioester bind is formed between the active site of E2 cysteine and C-terminal carboxyl group of Ub. Simultaneously, the E3 ligases interact with an Ub-loaded E2 enzyme and a substrate, resulting in the binding between the lysine amino group of the substrate and the C-terminal of carboxyl group of Ub, which is the final step of ubiquitin transfer [19] (Fig. 1).

Furthermore, the mechanisms of DUBs’ functions are illustrated. Firstly, through removing nondegradative ubiquitin signals, DUBs can make a difference to protein functions directly or influence multiprotein signaling complexes formation. Besides, the rescue of proteins from either proteasomal or lysosomal degradation caused by DUBs makes contributions to the protein homeostasis and signaling in cells. Moreover, DUBs have the capacity of maintaining free ubiquitin levels via recycling ubiquitin from degraded proteins and processing ubiquitin chain following its removal. Finally, DUBs can release monomeric ubiquitin from multimeric precursor proteins encoded by four genes, indicating its involvement in the process of newly synthesized ubiquitin generation [11].

Being one of the major degradation pathways, ubiquitin–proteasome system functions essentially in protein quality control system, during which substrates are initially ubiquitinated by the above enzymatic cascade. And after the recognition of ubiquitinated proteins, they are delivered to the proteasome. During substrate delivery, ub-binding receptors containing part of proteasome itself and others merely associating with it are required for proteasomal degradation of target proteins. Furthermore, the proteasome associated Ub receptors, such as Rpn10 and Rpn13, only uptake closely distanced poly-ubiquitinated substrates. While not being integral subunits of the proteasome, shuttling Ub-binding receptors can recognize ubiquitinated cargos situated far from the proteasome [20–22]. Moreover, it has been shown that in the delivery of poly-ubiquitinated substrates to the proteasome, the structure containing an N-terminal, Ub-like domain and a C-terminal Ub-associated domain of classical proteasomal shuttle proteins takes significant part [23].

### Crosstalk with other post-translational modifications

Not merely ubiquitination but also other reversible posttranslational modifications such as phosphorylation plays essential role in protein activity regulation. Single posttranslational modification has been demonstrated being capable of regulating protein functions, and multiple modifications gradually attract researchers’ concentration.

**Fig. 2:**
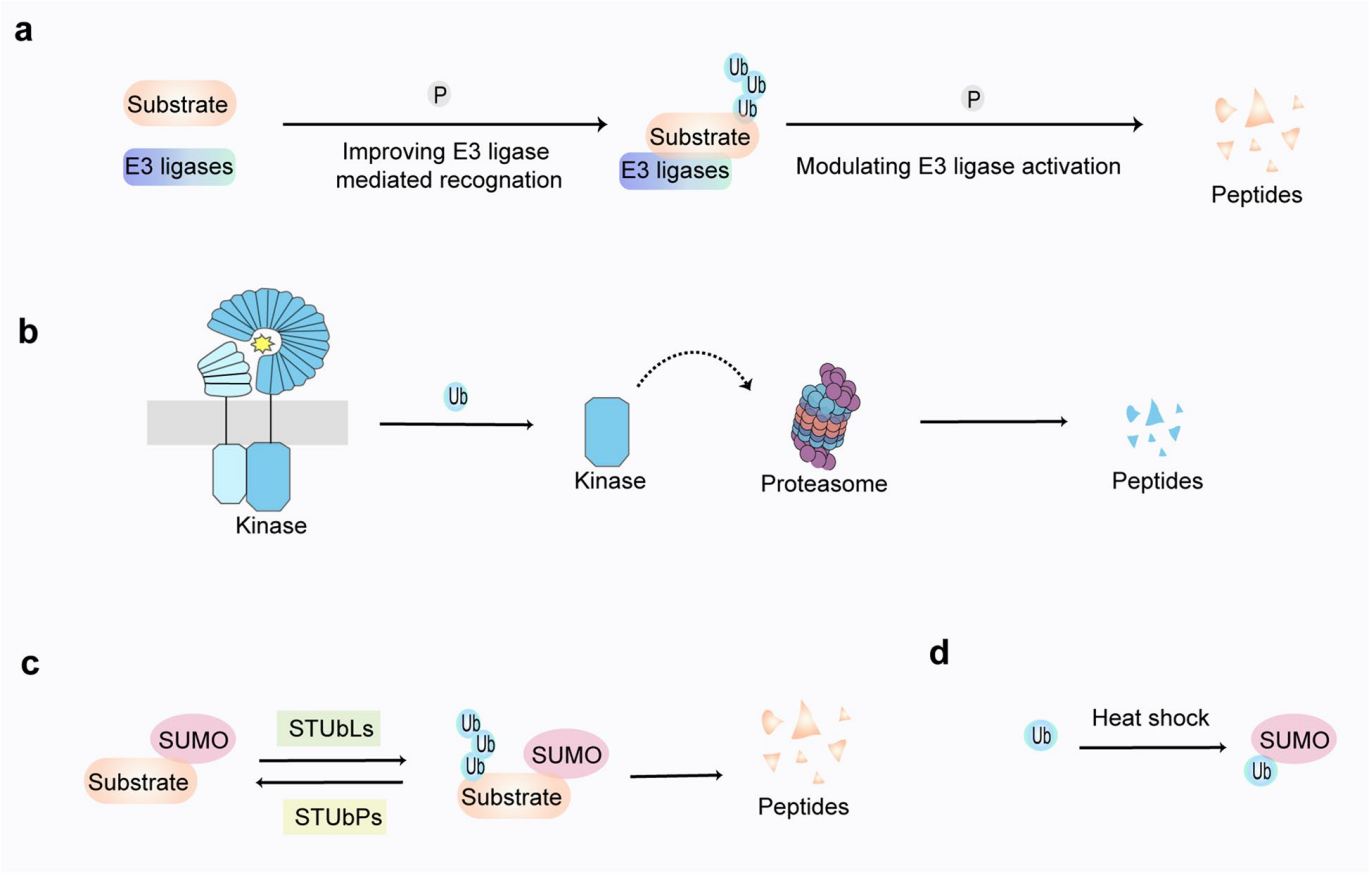
Crosstalk between posttranslational modifications. **a** During ubiquitination, phosphorylation can improve E3 ligase mediated recognition and modulate the activation of E3 ligases to influence the stabilization of proteins. **b** Many kinases can be degraded in ubiquitin-dependent manner when activation of protein kinases is sustained. **c** SUMO polymers function positively on ubiquitin–proteasome degradation of proteins, during which STUbLs mediate the ubiquitination of SUMOylated proteins and the process can be antagonized by STUbPs. **d** With heat shock and proteasome inhibition, ubiquitin can be SUMOylated although functions of SUMO-ubiquitin chains are unclear. Abbreviations: STUbLs, SUMO-targeted ubiquitin E3 ligases

Ubiquitination shares similarity with phosphorylation, including their engagement in various cellular processes, catalyzation caused by large numbers of transferases and reversion induced by large numbers of hydrolases. Study shown crosstalk between ubiquitination and phosphorylation occurs with the following manners (Fig. 2). First of all, phosphorylation can take positive or negative part in modulating activation of E3 ligases, and then through creating a phosphodegron, phosphorylation can improve E3 ligase mediated recognition. Moreover, at the level of subcellular compartmentalization, through impacting the association between substrate and ligase, phosphorylation makes a difference to ubiquitination. Additionally, under the activation of protein kinases including both Ser/Thr and Tyr kinases, especially when the activation is sustained, many protein kinases are subject to ubiquitin-dependent degradation. However, we can also observe the activation of kinases caused by ubiquitination such as TAK1 we will introduce below [24].

**Fig. 3:**
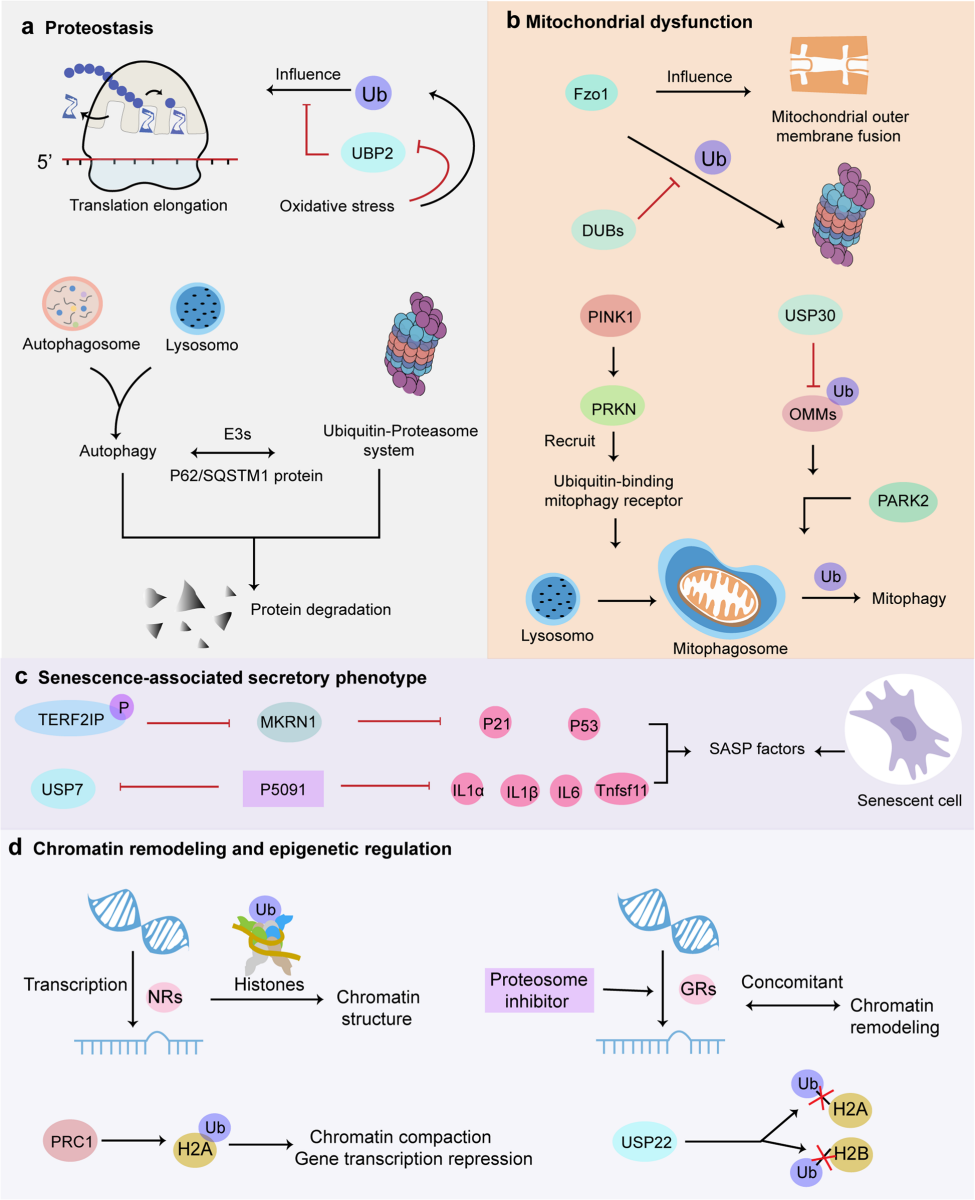
The main hallmarks of ageing ubiquitin system participate in. Ubiquitin proteasome system and DUBs participate in regulating ageing hallmarks including proteostasis, mitochondrial quality control, senescence-associated secretory phenotype, epigenetic regulation and genomic instability, indicating the association between ubiquitin system and ageing process. **a** For proteostasis, ubiquitination of ribosomes can control translation elongation under oxidative stress while UBP2 which regains functions with recovery from oxidative stress can alleviate the effect of ubiquitination. Moreover, the ubiquitin–proteasome system and the autophagy-lysosome pathway contribute to the degradation of proteins, and E3s and p62/SQSTM1 protein function as crosstalk between these two pathways. **b** For mitochondrial quality control, Fzo1, whose proteasome-dependent turnover depends on ubiquitination, can influence the fusion of mitochondrial outer membranes, and the ubiquitination can be reversed by UDBs. Furthermore, PINK1 can improve mitophagy mediated by PRKN, while USP30 results in counteraction of PARK-2 mediated mitophagy. **c** For senescence-associated secretory phenotype, ubiquitin E3 ligase MKRN1 can be downregulated by TERF2IP S205 phosphorylation, bringing about the upregulation of p21 and p53. And USP7 inhibitor P5091 can abrogate doxorubicin induced increase SASP factors involving Il1α, Il1β, Il6, and Tnfsf11. **d** For epigenetic regulation and genomic instability, ubiquitination of histones improves impact of NR-mediated transcription on chromatin structure. GR mediated transcriptional regulation is concomitant with chromatin remodeling and transcriptional ability of the GR can be enhanced by proteasome inhibitors. Moreover, DUBs including PRC1 and USP22 can make a difference to the ubiquitination of H2A and H2B

Being members of the ubiquitin-like family of proteins, small ubiquitin-like modifiers (SUMOs) can connect with quantities of proteins. In the process of the target protein degradation mediated by the ubiquitin–proteasome system, SUMO polymers play a positive role, during which SUMO-targeted ubiquitin ligases (STUbLs) mediate the ubiquitination of SUMOylated proteins and subsequent degradations by the proteasome [25] (Fig. 2). While the process can be counteracted by SUMO-targeted ubiquitin proteases (STUbPs), leading to the deubiquitination of SUMOylated proteins. And the best known STUbP is USP7, contributing to the removal of the ubiquitin from SUMOylated proteins to maintain the SUMO-rich and ubiquitin-poor environment required for DNA replication [26]. Besides the ubiquitinated SUMO chains, researchers also demonstrate the ubiquitin can be SUMOylated and in response to heat shock and proteasome inhibition, SUMOylation of ubiquitin Lys26 and Lys27 are upregulated. However, the functional roles of the SUMO-ubiquitin chains type are still under explored [27]. Furthermore, we have illustrated before that SUMO can inhibit the activation of USP28 through direct interaction with the catalytic domain of USP28 [28]. All we have discussed above shown the mechanisms of ubiquitination and interaction between diverse posttranslational modifications, showing sophisticated mechanisms in cellular functions, and we will then illustrate the influences of ubiquitination on ageing based on cellular ageing and homeostasis (Fig. 3).

## Ubiquitin system in cellular ageing and homeostasis

### Proteostasis and protein quality control in ageing cells

Protein homeostasis, referring to the state of a balanced proteasome, requires the proteins maintaining folded to perform their biological functions and the abundance of proteins remaining controlled in a mammalian cell [29]. To achieve protein homeostasis, the proteostasis network is indispensable to protect from the accumulation of potentially toxic protein aggregates. However, it is illustrated that proteostasis is disturbed in many pathological conditions especially diseases associated with old age, suggesting the negative role of ageing in maintain the capacity of the proteostasis network [30].

Furthermore, the association between mitochondria and ageing process is also demonstrated. In the production process of energy required by the cell, mitochondria take crucial part. And the energy is provided by the electron transport chain, while reactive oxygen species (ROS) are produced at the same time. ROS are known for its damage on proteins especially mitochondrial proteins and protein damage results in mitochondrial function impairment. Thus, mitochondrial have their own protein quality control system to cope with this situation [31, 32]. The accumulated damage proteins and dysfunctional mitochondrial have been illustrated making contributions to ageing and age-related diseases [33, 34].

### Ubiquitin system in oxidative stress responses and mitochondrial quality control

Excessive ROS or their derivatives contribute to oxidative stress, resulting in the detriment of cells and organisms. Containing antioxidants and antioxidant enzymes and proteolytic systems, varieties of protection mechanisms are developed to address oxidative stress in organisms. Furthermore, an intricate regulatory network of gene expression at both the transcriptional and translational levels can also be evoked. Study shown that via ubiquitination of ribosomes, translation elongation can be controlled during oxidative stress based on lysine K63 linked ubiquitin monomers [35, 36]. Moreover, during the process of oxidized protein degradation, certain oxidized proteins prefer ubiquitination and proteasome inhibition leads to the accumulation of ubiquitinated species of oxidized proteins [37]. Proteasome inhibitors have the ability to maintain the levels of ubiquitinated oxidized proteins and during the recovery from mild oxidative stress, transient increases of ubiquitinated protein level can be seen correlated with increased intracellular proteolysis [38].

Besides of ubiquitination and proteasome, DUBs also participate in oxidative stress responses. For instance, DUB UBP2 can be reversely inhibited through oxidation and during the recovery from oxidative stress, oxidation inhibited UBP2 rapidly regains function. Then UBP2 removes K63-linked ubiquitin from ribosomes and alleviates its effect on translation. In the pathway, as a redox sensor, DUB UBP2 promotes reduction of translation required for appropriate stress response [35]. All above indicates the involvement of ubiquitin system in oxidative stress responses.

Damaged mitochondrial proteins can be targeted to proteasomal degradation in the cytosol via ubiquitin dependent pathway to maintain protein control. In addition to involvement in damaged mitochondrial protein degradation, ubiquitin–proteasome system also plays vital role in the remodeling of mitochondrial proteome [39]. For example, ubiquitination of the first identified MAD substrate the yeast mitofusin Fzo1 makes a difference to the fusion of mitochondrial outer membranes. Besides, proteasome-dependent turnover of Fzo1 largely relies on the nature of ubiquitination, which can be reversed by DUBs [40, 41].

### Regulation of mitophagy and autophagy via ubiquitin system

As we have shown above, in the process of mitochondrial proteins assembling, errors in importing and folding result in the damage of mitochondria, which is essential for cells and organisms. Unreversible damage contributes to the elimination of mitochondria via mitophagy. During the process of targeting mitochondrial to the autophagy system, the fundamental biological steps of mitophagy engaged are ubiquitin-dependent and ubiquitin-independent.

And Parkinson disease-the E3 ubiquitin-protein ligase parkin (PRKN) and its activating kinase PTEN-induced putative kinase 1 (PINK1) are critical in the ubiquitin-dependent mitophagy [42, 43]. In healthy mitochondria, PINK1 fragment can be released into the cytosol and ubiquitinated, contributing to the its degradation. While in damaged mitochondria, accumulated PINK1 promotes the E3 ubiquitin ligase activity of parkin, leading to the improvement of ubiquitin-binding mitophagy receptor recruiting. Moreover, PINK1 mediates the phosphorylation of ubiquitin, enhancing the activation of parkin and mitochondrial retention [44].

As for the DUBs’ functions in mitophagy, study has shown that both USP30 and USP35 participate in the modulation of PARK2-mediated mitophagy. Previously, three outer-mitochondrial membrane (OMM) proteins including MFN2, VDAC1 and TOMM20 have been demonstrated to be degraded under the activation of PARK2-mediated mitophagy [45]. During mitophagy, USP30 can regulate the levels of ubiquitinated MFN2 and TOMM20, contributing to the counteraction of PARK2 activity and the delay of PARK2-mediated mitophagy. Moreover, researchers assume that USP35 can maintain the levels of mitochondria morphology proteins such as MFN2 and play a housekeeping role in healthy mitochondrial, although the underlying mechanisms of USP35’s impacts on mitochondrial quality control are still obscure [46].

Furthermore, in the protein quality control being crucial in proteostasis, protein degradation mainly depends on two pathways, the ubiquitin–proteasome system and the autophagy-lysosome pathway. Autophagy contains macroautophagy, microautophagy and chaperone-mediated autophagy and it is a cytoprotective pathway, functioning on the cytosolic component delivery to the lysosomes for degradation [47–49]. Although UPS and autophagy are different mechanisms of degradation, they share some molecular players including ubiquitin, E3s and p62/SQSTM1 protein, a significant crosstalk between UPS and autophagy. The UPS can regulate the activity of autophagy-lysosome pathway via transcription factors modulation and autophagy can influence UPS through clearance of proteasomes. Besides, investigations shown with deficient degradation of ubiquitinated proteins induced by UPS such as upon proteasome inhibition, autophagy can be upregulated. The coordination of UPS and autophagy results in a coordinated response against environment cues including oxidative stress, while the crosstalk between UPS and autophagy is attenuated with ageing [50].

Furthermore, under well-fed conditions, knockdown of Leon/USP5 contributed to the remarkable increase of the formation of autophagosomes and autophagic flux. Further immunoblotting assays shown a strong interaction between Leon/USP5 and the autophagy initiating kinase Atg1/ULK1 and depletion of Leon/USP5 resulted in the increase of Atg1/ULK1, indicating the negative role of Leon/USP5 in the autophagic process. However, the underlying mechanisms of the interaction still need further investigations [51]. Besides, depletion of dUSP36 contributed to the inhibition of cell growth while activated autophagy and the induction of autophagy depends on p62/SQSTM1 [52]. All we have discussed above indicates the involvement of ubiquitin system in autophagy and mitophagy.

### Influence on senescence-associated secretory phenotype (SASP)

Additionally, varieties of hallmarks containing genomic instability, telomere attrition and cellular senescence can propel ageing at the cellular level. Being faced with stressors such as DNA damage, oxidative stress and oncogene activation, cells will enter in a state of irreversible growth, which is referred to be as cellular senescence. And senescent cells can produce pro-inflammatory cytokines, chemokines, growth factors and matrix-degrading enzymes, whose mixture is known as senescence-associated secretory phenotype, a major factor link between senescence and aging pathology [53]. Previous investigation has shown that in endothelial cells, MKRN1 ubiquitin E3 ligase downregulation caused by TERF2IP S205 phosphorylation can contribute to the promotion of senescence and inflammation by upregulation of p21 and p53, suggesting the influence of ubiquitination on SASP [54]. Moreover, in p16-3MR mice treated with doxorubicin, treatment with USP7 inhibitor P5091 can not only eliminate senescent cells but also abrogate doxorubicin induced increase SASP factors involving Il1α, Il1β, Il6, and Tnfsf11 [55], indicating the influences of DUB on SASP.

### Ubiquitin system in chromatin remodeling and epigenetic regulation

Changing of the epigenome is one of the crucial mechanisms during ageing and in age-related disorders, chromatin remodelers can modulate gene expression in response to the changes of nutrient environments and energy metabolism [56]. In the transcriptional control caused by nuclear hormone receptors (NRs), the organization of chromatin is of fundamental significance. Post-translational modifications of histones involving phosphorylation and ubiquitination play a positive role in the impacts of NR-mediated transcription on chromatin structure [57–59]. Besides, ubiquitin-proteosome system components can function on the regulation of NR-mediated transactivation due to their direct modification on chromatin. Moreover, transcriptional regulation caused by glucocorticoid receptors is concomitant with chromatin remodeling and we can observe increased transcriptional ability of the GR with the presence of proteasome inhibitors in the absence of elevated chromatin remodeling, suggesting the involvement of the ubiquitin–proteasome system components in the downstream of transcriptional initiation [60]. All above illustrate the association of UPS and chromatin remodeling.

Besides, brief introductions of DUBs’ involvement in epigenetic regulation are given. Polycomb repressive complex 1 (PRC1) has the capacity of monoubiquitinating H2A histone, contributing to the compaction of the chromatin and then repression of gene transcription [61]. Researchers shown that USP7 is a regulator of PRC1 [62]. Moreover, USP7 can influence EZH2, a functional component of the PRC2 [63]. USP22 can remove the monoubiquitin in lysines 119 and 120 through interacting with H2Aub1 and H2Bub2 (ubiquitination marks in H2A and H2B) [64, 65], indicating the functions of DUBs in regulating chromatin structure.

## Roles of DUBs in ageing-related diseases and processes

Just as we have discussed above, ubiquitination plays a significant role in cellular ageing and homeostasis, directing our further investigations into the relationship between ubiquitination and ageing-related diseases, among which deubiquitinases are of significance. Among all of the families of DUBs, ubiquitin-specific protease (USP) is the biggest one [66]. The structure of various USPs’ ubiquitin-binding domains have been revealed, including the zinc finger ubiquitin-specific protease (ZnF-UBP) domain, the ubiquitin-interacting motif (UIM) and the ubiquitin-associated (UBA) domain [66].

Based on distinctive modular domain architecture, DUSP-containing USPs are grouped into clusters. Among these USPs, USPs 20 and 33 are homologous with split catalytic USP domains, and each of enzymes contains a pair of tandem DUSPs following the catalytic domain [67, 68]. Both USP20 and USP33 can interact with pVHL and be ubiquitinated in the pVHL-dependent manner [69]. And the interactions of DUBs and E3 function diversely, among which the association between USP20 and pVHL allows the E3 to regulate the target and its DUB simultaneously [70]. Furthermore, researches shown that USPs 20 and 33 have redundant functions and one of the enzymes is able to compensate for the absence of the other [71, 72].

Here, we pay our attention to the association between DUBs, especially USP20, an emerging DUB which can function on a number of substrates and then reverses the ubiquitination, and ageing-related diseases to describe the close relationship between UPS and ageing (Table 1). Moreover, we will also involve some other DUBs’ functions during the illustration.

**Table 1:** Effects of DUBs in diverse aging-related diseases and processes

Categories of diseases and process	Type of diseases and process	Related DUB	Related target proteins	Clinical role of DUB in the diseases	References
Cancer	Breast cancer	USP20	SNAI2; ERK3; β-catenin	Higher level of USP20 correlates with worse relapse-free survival and metastasis-free survival and results in chemoresistance to cisplatin and etoposide	[73–75]
		USP20	PD-L1	Inducing breast cancer immune escape and disease progression	[76]
	Colorectal cancer	USP20	β-catenin	-	[75]
		USP20	SOX4	-	[77]
		USP20	-	High USP20 expression associates with lymph node metastasis, chemotherapy resistance and a short OS	[78]
	Lung cancer	USP20	β-catenin	USP20 contributes to the chemoresistance	[75]
		USP20	Claspin Rad17	-	[79–81]
		HAUSP	P53	HAUSP functions as a tumour suppressor	[82]
	OSCC	USP20	STING	USP20 results in the function of OVT and its inhibitor GSK2643943A enhances the therapy of oncolytic HSV-1	[83]
	HNSCC	USP20	CTSL	Contributing to chemoresistance in HNSCC	[84]
	Oesophageal cancer	USP20	MCL1	USP20 overexpression decreases the toxicity induced by the combination of Sorafenib and ABT-263	[85]
	Hepatocellular carcinoma	USP20	SLC7A11	USP20 overexpression contributes to OXA resistance and poor prognosis	[86]
	Gastric cancer	USP20	Claspin	Lower level of USP20 correlates with bigger tumor size, more tumor invasion, late TNM staging and worse prognostic	[87]
	Bladder cancer	USP20	YAP1	Higher expression of USP20 correlates with poor prognosis	[88]
	T-ALL	USP20	HIF1A	USP20 is a promising target in T-ALL treatment	[89]
	ATL	USP20	Tax	-	[90, 91]
	PDAC	USP20	HMGCR	Combination of USP20 inhibitors and anti-PD-1/anti-CTLA-4 immunotherapy results in better anti-tumour efficacy	[92]
	Glioblastoma	USP15	Type I TGF-β receptor	USP15 amplification confers poor prognosis in individuals with glioblastoma	[93]
Neurodegenerative Diseases	Parkinson's disease	UCH-L1	α-Synuclein	UCHL1 variant I93M can increase risk of PD while S18Y functions as a protective factor of PD	[94]
		USP8	α-Synuclein	USP8 is a promising therapeutic and diagnostic target for PD	[95]
		USP30	α-Synuclein	USP30 inhibitors are the potential therapy for PD	[96]
	Alzheimer's disease	USP14	Tau; TDP43; ATXN3	-	[97]
	Huntingtin’s disease	USP12	-	UPS12 helps to further establish the therapeutic potential of this genetic modifier for the treatment of HD	[98]
Cardiovascular diseases	Heart failure	USP20	-	Carvedilol improves heart failure through inhibiting the binding of USP20 and β_2_AR	[71, 99, 100]
		USP20	HIF1α	-	[101]
		USP20	STAT3	-	[102]
	Ischemic stroke	USP20	-	USP20 can attenuate cerebral ischemic strokes and ameliorate cognitive impairments	[103]
	Atherosclerosis	USP20	RIPK1	USP20 weakens the atherogenic signaling	[104]
	Septic cardiomyopathy	USP20	NLRP3	-	[105]
	-	USP10	Beclin 1	Acacetin can inhibit USP10 and is a promising candidate drug for the treatment of cardiac remodeling induced by pressure overload	[106]
Metabolic diseases	Metabolic disease	USP20	HMGCR	USP20 inhibitor GSK2643943A contributes the reduction of weight while promotes thermogenesis	[107]
	Osteoporosis	USP20	FTO	-	[108]
	Obesity	USP20	HSPA2	SG contributes to decrease of USP20 and then reduces lipid accumulation	[109]
		USP19	-	USP19 depletion results in body weight reduction	[110]
		USP2, 7, 15 and 19	-	Contributing to obesity	[111]
		USP53	-	USP53 might be restorative for obesity	[111]
Immune response	Viral infection	USP20	STING	Depletion of USP20 results in being more susceptible to HSV-1 infection	[112]
	NF-κB related inflammatory	USP20	TRAF6; β-arrestin2 RIPK1	USP20 can attenuate the neointimal hyperplasia caused by vascular inflammation	[104, 113]
		USP20	P62	-	[114]
	-	USP25	TRAF6	-	[9]
	-	USP44, CYLD	STING	-	[9]
	Chronic inflammatory and autoimmune diseases	USP4	RORγt	USP4 could be a novel therapeutic target for the treatment of Th17-modulated autoimmune diseases	[115]
Autophagy	Autophagy	USP20	ULK1	Reducing the apoptotic cell death under starvation	[116]
		USP20	RETREG1	-	[117]
		USP33	PRKN	-	[118]
		USP10	GSK3β	-	[119]

*Abbreviations ATL* Adult T cell leukemia, *ATR* Ataxia Telangiectasia and Rad3-related, *Chk1* Checkpoint kinase 1, *ERK3* Extracellular signal-regulated kinase 3, *HSV-1* Herpes simplex virus type 1, *IFN* Type I interferon, *IL-1β* Interleukin-1β, *MCAO* Middle cerebral artery occlusion, *MCL1* Myeloid cell leukemia 1, *mTORC1* mechanistic target of rapamycin complex 1, *NF-κB* Nuclear Factor kappa B, *OSCC* Oral squamous carcinoma cell, *OVT* Oncolytic virotherapy, *PTEN* Phosphatase and tensin homolog deleted on chromosome 10, *RETREG1* Reticulophagy regulator 1, *RIPK1* Receptor-interacting protein kinase 1, *SG* Sleeve gastrectomy, *SMC* Smooth muscle cell, *SOX4* SRY-related HMG-box 4, *STING* Stimulator of interferon genes, *TLR4* Toll-like Receptor 4, *TNFR* Tumor necrosis factor receptor, *TRAF6* Tumor necrosis factor receptor-associated factor 6, *ULK1* Uncoordinated 51-like kinase 1, *USP20* Ubiquitin-specific protease 20, *VCAM1* Vascular cell adhesion molecule

### Roles of DUBs in cancers

Through modulating different hallmarks of cancer such as cell proliferation, angiogenesis, metastasis, USP20 makes a difference to diverse cancers. Other than nonmelanoma skin cancer, breast cancer is the most common cancer for women around the world [120]. And with the age acceleration, the risk of breast cancer is rapidly growing [121]. In breast cancer cells, knockdown of USP20 or SNAI2, which is known to promote cell migration and invasion and one of the substrates of USP20, can contribute to the attenuation of invasive capabilities. Meanwhile, the consequence caused by the depletion of USP20 can be rescued by the overexpression of SNAI2, indicating that the functions of USP20 on the cancer is displayed via SNAI2. And compared with the control cells in *vivo* experimental lung colonization studies, results shown less cells seeded in lung and less metastasis nodules formed without USP20 or SNAI2 [73]. Besides, USP20 can deubiquitinate both WT and lysine-less ERK3 proteins in cells with the mediation of its interaction with N-terminal kinase of ERK3. In HeLa cells, USP20 overexpression results in the similar effect with ERK3 through inducing the reorganization of actin cytoskeleton and promoting cell migration in an ERK3-dependent manner, and the inhibition of cell migration induced by USP20 silence can be rescued by ERK3 overexpression in MCF7 and MCF10A cells [74, 75].

Furthermore, β-catenin, the central mediator of the Wnt/β-catenin signaling pathway, can be ubiquitinated and then degraded mediated by the function of the E3 ligase β-TrCP, which can be antagonized by USP20 in both APC-GSK3β-β-TrCP-dependent and -independent manner. And the enzymatic domain of USP20 and the N-terminus of β-catenin containing 1–97 amino acids---UCH domain is in requirement in the association between USP20 and β-catenin. In both A549 cells and other cell lines including U2OS, Ovsar7 and Colo 205, significantly decreased protein levels of β-catenin and its target gene TCF1/TCF7 and MMP-7 can be seen with depletion of USP20, indicating the regulation of β-catenin pathway caused by USP20. Through stabilization of β-catenin, USP20 promotes cancer cell growth, tumorigenesis and cancer cell invasion and migration. And overexpression of USP20 can bring about the occurrence of resistance to cisplatin and etoposide in A549 and MDA-MB-231 cells while this can be turned over by the knockdown of β-catenin [75]. In accordance with the impacts of USP20 in breast cancer, higher level of USP20 correlates with worse relapse-free survival and metastasis-free survival in patients suffering breast cancer [73].

Besides, a novel study has demonstrated that in breast cancer, LncRNA tissue differentiation inducing non-protein coding RNA (TINCR) can recruit USP20 and then leads to PD-L1 upregulation, impairing the immunotherapy against breast cancer. In the investigation, expression of TINCR shown in both the cytoplasm and nucleus of breast cancer cells. In the cytoplasm, TINCR functions as a molecular sponge for miR-199a-5p, and through the ceRNA regulatory mechanism the stability of USP20 mRNA is upregulated, contributing to the promotion of the expression of PD-L1 by inhibiting its ubiquitination. Besides, in the nucleus, TINCR recruits DNMT1, which can promote methylation and then inhibit the transcription of miR-199a-5p, decrease of which weakens its inhibition of USP20 mRNA stability in the cytoplasm, resulting in the upregulation of PD-L1 expression. Consequently, the upregulation of PD-L1expression has the capacity of inducing breast cancer immune escape and promoting disease progression [76]. Moreover, USP20 takes a part in colorectal cancer by deubiquitinating EMT transcription factor SOX4 and promoting lymph node metastasis. USP20 not only functions as an independent risk factor for poor prognosis but also takes part in drug resistance in patients suffering colorectal cancer [77, 78].

HIF-1α can enforce the progression of cancers via affecting angiogenesis, cell survival and invasion. USP20 can connect to HIF-1α in a pVHL-dependent manner and turn over its ubiquitination to stabilize it [122]. Study shown that it’s USP20 rather than USP33 that interacts with HIF-1α because the HIF-1α-binding region in USP20, situating in amino acids 269–390, shares less sequence homology with the same region in USP33. Meanwhile, although USP20 can also be ubiquitinated by pVHL, the increasing level of HIF-1α is caused by the catalytic activation of USP20 instead of the competition between the two substrates in binding to pVHL [123]. In prostate cancer whose risk rises with age [124], hSP56 can interact with USP20 and it has the ability to inhibit HIF-1α [122, 123], bringing about the suppression of the cancer. Nevertheless, whether there exists relationship between the interaction of USP20 and heat shock protein 56 (hSP56) and the inhibition of HIF-1α caused by hSP56 still remains unknown [125].

Moreover, the Hippo-YAP1 axis is inhibited in bladder cancer (BC), which is a major driver of BC progression and oncogenesis and study conducted in bladder cancer shown USP20 directly interacts with Yes-associated protein 1 (YAP1) and increases its stability by removing the K48-linked polyubiquitin chains from YAP1. In BC cells, knockdown or depletion of USP20 results in induced cell cycle arrest, increased apoptosis and reduced lung metastasis, while all of which can be reversed by YAP1 overexpression. USP20 expression is elevated in different human cancers and is associated with poor survival [88].

An epidemiology study concerning oral tongue squamous cell carcinoma revealed that compared with older patients, improved survival rates are observed in younger patients [126]. Oncolytic virotherapy (OVT) is an immunotherapy in which viruses preferentially kill tumor cells and induce antitumor immune response of the body and it can be applied to oral squamous carcinoma cell (OSCC) [127, 128]. STING is significant in the innate anti-viral signaling, and its stabilization can be enhanced by USP20 and USP18. Study shown that oncolytic HSV-1 T1012G replication increased when USP20 and USP18 were knocked down, and this process was mediated by the reduction of STING. That may be the reason for the different sensitivity of OVT towards diverse human OSCC lines, in which USP18 and USP20 are accumulated with different levels. Furthermore, treating SCC9 cells which possess a higher level of USP20 and USP18 with USP20 inhibitor GSK2643943A and oncolytic HSV-1 T1012G shown a profound anti-tumor effect. The results illustrate that USP20 can be a reason for immunotherapy resistance due to its acceleration in anti-viral response of the body [83]. In addition, in comparison with the normal oesophageal tissues, USP20 and its substrate MCL1 display higher expression in oesophageal cancer. The overexpression of USP20 can also weaken the toxicity of the combination of Sorafenib and ABT-263 towards the ECA-109 cells [85].

Besides, cathepsin L (CTSL), a lysosomal cysteine protease, is increasingly implicated in EMT and tumour progression in multiple cancers including head and neck squamous cell carcinoma (HNSCC) [129, 130]. Guo et al. demonstrated that in head and neck squamous cell carcinoma, USP20 can competitively bind STUB1, contributing to the inhibition of STUB1-mediated ubiquitination and degradation of cathepsin L. Moreover, USP20 possesses the capacity of removing the K48-linked ubiquitin chains, resulting in the stabilization of CTSL. With the stabilization of CTSL, CTSL-driven EMT, CSC self-renewal and metastasis in HNSCC are promoted, and chemoresistance is also conferred [84], indicating the targetable potential in HNSCC therapy.

In oxaliplatin (OXA)-resistant hepatocellular carcinoma cells we can observe high levels of ATR, USP20 and SLC7A11 (an antiferroptosis protein). DNA damage‐induced ATR activation is required for Ser132 and Ser368 phosphorylation of USP20. Phosphorylation of USP20 caused by ATR increases the protein level of USP20. UCH domain of USP20 can physically bind to the N-terminal of SLC7A11 and during the process of deubiquitination, K30 and K37 of SLC7A11 are the key sites. The stabilization of SLC7A11 then protects cells from ferroptosis through the Xc −/GSH/GPX4 axis pathway. These results suggest that OXA induced DNA damage leads to USP20/SLC7A11‐dependent ferroptosis resistance in HCC cells, indicating USP20 is a contributor to OXA resistance and can suppress ferroptosis in HCC. And in the study, compared with the administration of either USP20 inhibitor GSK2643943A or OXA alone in vivo tumorigenesis, administration of combination of GSK2643943A and OXA promotes the decrease of tumor growth. Therefore, USP20 correlates with OXA resistance and functions as an adverse biomarker for HCC patients’ prognosis [86].

Aging is not only the risk factor for most forms of lung cancer, it’s also positively related to the incidence rate for gastric cancer [131, 132]. Besides the oncoprotein β-catenin discussed above is stabilized by USP20, study has also exposed that USP20 can deubiquitinate Rad17 and claspin which are closely associated with the activation of DNA replication checkpoint in A549 cells [79–81]. The protein complex composed of Rad17 and the Replication Factor C (RFC) subunits makes contributions to the interaction of the PCNA-like Rad9-Hus1-Rad1 (9–1-1) clamp with the DNA ends at the arrested fork. And as the activator of ATR which mediates the phosphorylation of Chk1, topoisomerase-binding protein-1 (TopBP1) is recruited by 9–1-1 clamp [133]. Mediated by more than one domain, USP20 can associate with Rad17, and among the diverse association the strongest binding is mediated by the USP20 N-terminus, resulting in the stabilization of Rad17. Besides, as we discussed above, through phosphorylation, Rad17 can facilitate Chk1 activation under DNA damage response and we can observe that after USP20 depletion the forced expression of Rad17 can rescue the decrease of Chk1 phosphorylation [79].

Moreover, after the treatment of Hydroxyurea (HU) or ultraviolet (UV), we can see that USP20 ubiquitination is dramatically decreased. And the N-terminus of claspin (1–330 AAs) mediates the interaction with USP20, contributing to the removal of K48-linked polyubiquitination chains of claspin and its stability. Further investigation shown that under genotoxic stress, HERC2, an E3 ubiquitin ligase of USP20, disassociates from USP20, resulting in the upregulation of USP20 and its substrate claspin. During the process, ATR-mediated phosphorylation of USP20 can induce the disassociation of HERC2 and USP20, which is in requirement for USP20 stabilization. In conclusion, USP20 has a positive function on the regulation of Rad17-claspin-Chk1 signaling and maintenance of the genome stability in replication stress, indicating that USP20 is a tumor suppressor in lung cancer [79–81].

Moreover, PLK1 is in requirement for the recovery from the checkpoint which is activated by Chk1 after DNA damage. And the depletion of USP20 can result in the decrease of PLK1, which can explain the reason for the absence of checkpoint-defect in USP20-depleted cells [79]. Not only in lung cancer, USP20 and claspin also have a positively correlated expression in gastric cancer, and both are lower than in peritumoral gastric tissues. And higher expression of USP20 and claspin predict better prognosis in patients suffering from gastric cancer [87].

As one of the retrovirus, HTLV-1 takes positive part in adult T cell leukemia (ATL), a fatal hematopoietic malignancy [134]. Being a transactivator of both viral and cellular genes, the HTLV-1 Tax oncoprotein functions critically in the initiation of leukemogenesis and it can be deubiquitinated by USP20 [90, 91]. Ubiquitinated Tax is indispensable for the activation of NF-κB and Tax-NF-κB pathway are important for cellular transformation induced by HTLV-1. Furthermore, in the ATL2 cells transfected with USP20, slowing down of cell proliferation occurred distinguishably, which indicates that USP20 has the capacity of inhibiting the progression of ATL [90].

Besides, T-ALL is a highly aggressive form of leukemia characterized by the clonal proliferation of abnormal T lymphocytes with poor treatment response, higher relapse rates, increased risk of progression to refractory leukemia. Study shown that knockdown of USP20 contributes to increased T-ALL cell apoptosis and inhibited cell proliferation and in mice *USP20* knockdown reduced tumor growth and prolonged survival. The results are achieved through the deubiquitination of HIF1A by inhibiting K48-linked polyubiquitination induced by USP20 and regulation of gene transcription through HIF1A. And similar to knockdown of USP20, GSK2643943A, USP20 inhibitor, inhibited the proliferation and progression of T-ALL, indicating USP20 as a promising target in T-ALL treatment [89].

Except for the underlying mechanisms discussed above concerning the influences on diverse cancers, Jiang et al. shown the USP20-driven cholesterol metabolism links inflammatory signaling to malignancy and stromal co-evolution in pancreatic cancer [92]. The investigation demonstrated that in tumour cells, TNFSF13B^+^ macrophage-activated STAT3 inflammatory signaling induces USP20 transcription, contributing to the protection of HMGCR from proteasomal degradation. HMGCR deubiquitination results in the promotion of mevalonate metabolism and YAP/TAZ signaling, thus improving tumour cell proliferation and triggering the activation of cancer-associated fibroblasts. Moreover, in pancreatic ductal adenocarcinoma, combination of USP20 inhibitors and anti-PD-1/anti-CTLA-4 immunotherapy led to better anti-tumour efficacy [92].

Except for the diverse influences caused by USP20 in varieties of cancers, we can also observe the relationship between other DUBs and cancer-related molecules. For instance, USP15 can make a connection with the SMAD7–SMAD-specific E3 ubiquitin protein ligase 2 (SMURF2) complex, and deubiquitinate and stabilize the type I TGFβ receptor, leading to the enhancement of TGFβ signaling. Amplification of the *USP15* gene can be seen in glioblastoma, breast and ovarian cancers, and higher level of USP15 is correlated with higher TGFβ activity [93]. And DUBs involving USP7, the inhibitor of MDM2, have been demonstrated to suppress the degradation of tumor suppressor protein p53, which plays a key role in response to cellular stress and is lost or mutated in many cancers [82], indicating the important functions of DUBs in diverse mechanisms of progression of cancers.

### Roles of DUBs in neurodegenerative diseases

Alzheimer’s disease and related neurodegenerative diseases are referred to be as the most terrifying diseases of older people and neurodegeneration related characteristic changes can be observed in almost all aged brains [135]. We will give brief introductions of the association between DUBs and neurodegenerative diseases. Emerging researches have shown that cell-to-cell transmission of misfolded preformed fibrils (PFF) of α-synuclein (α-syn) may be the underlying mechanism of Parkinson's disease (PD) [136]. UCHL1 is a significant DUB in the brain, and it has been shown that UCHL1 colocalizes with α-syn. Furthermore, UCHL1 functions both as a deubiquitinase and a ligase which can extend Lys63-polyubiquitin chains on α-syn. And the ligase activity can be enhanced by oligomerization while disrupted by disease-associated mutations. UCHL1 variant I93M can increase risk of PD by disrupting activity, while by reducing dimerization and ligase activity, S18Y functions as a protective factor of PD [94].

Besides UCHL1’s functions on PD, polymorphic USP8 allele (USP8D442G) was discovered significantly enriched in Chinese PD patients through genome-wide sequencing. D442G polymorphism can enhance the interaction between α-Syn and USP8 and then increase the K63-specific deubiquitination and stability of α-Syn, contributing to accumulation and abnormal subcellular localization of α-Syn in dopaminergic neurons [95]. Moreover, knockdown of USP30 in mice leads to increased mitophagy, decreased phospho-S129 αSyn, and then attenuates dopaminergic neuronal loss induced by αSyn, indicating USP30 inhibitors as the potential therapy for PD [96].

Besides the DUBs discussed above, investigation has shown the association of USP14 and Alzheimer's disease. Accumulation of several proteins including Tau, TAR DNA-binding protein 43 (TDP43) and ATXN3 is closely associated with neurodegenerative diseases. It is shown in a study that the reversible USP14 inhibitor IU1 can target the USP14 catalytic site and promote degradation of overexpression of these proteins [97]. Moreover, USP12 can protect against polyglutamine‐expanded mutant huntingtin toxicity and enhance autophagy in neurons, resulting in the amelioration of Huntingtin’s disease [98].

### Roles of DUBs in cardiovascular diseases

Beta-adrenergic receptors (βARs) can be activated by their endogenous ligand norepinephrine, contributing to the increased workload of the heart. And then myocyte death as well as maladaptive cardiac remodeling closely related to the heart failure may occur [137]. And in the process of aging, heart failure is becoming more and more susceptible due to the deterioration of the cardiac structure and function [138].

Stimulation on β_2_AR caused by isoproterenol (Iso) resulted in the ubiquitination and lysosomal trafficking of the receptor while the overexpression of USP20 and USP33 could antagonize this process. And the antagonization of the process contributes to the acceleration of the receptor recycling from the late-endosomal compartments as well as resensitization of recycled receptors at the cell surface. Before agonist-stimulation and β-arrestin translocation, USP33 is bound to the receptor. With Iso stimulation, β-arrestin2 can recruit Nedd4 and its adaptor might function on removing the DUB from the activated β2AR, contributing to the improvement of receptor ubiquitination. Besides, β2AR activation induces changes in β-arrestin2 conformation, facilitating the association between β-arrestin2 and USP33 and then the deubiquitination of β-arrestin2. Sequentially, the disassembly of β2AR-β-arrestin2 signaling complexes are induced. Investigators think that when both proteins are in specific ‘activated’ conformations, there is a dynamic exchange of protein partners between the β2AR and β-arrestin [71].

Additionally, under nutrient starvation, synchronized by dynamic posttranslational modifications of USP20, agonist-activated ubiquitinated β2ARs traffic to autophagosomes, and PTMs of USP20 are induced in a β2AR-dependent manner in turn. Furthermore, the agonist-stimulated βAR induces the activation of PKAα rapidly, which then phosphorylates USP20 at serine 333. And USP20 phosphorylation at serine 333 results in its deubiquitinase activity blockage, accelerating its dissociation from the activated β2AR complex and the ubiquitinated β2AR traffic to autophagosomes [99]. USP20 functions significantly in the recycling of β_2_ARs in the experiments discussed above.

The phosphorylation of USP20 induced by the activation of β_1_AR is also mediated by PKAα, while what is contrary to the impact of the posttranslational modification mentioned before is that agonist-activated β_1_AR forms a stable complex with USP20, suggesting that phosphorylated USP20 associates robustly with the β_1_AR and facilitates the deubiquitination of β_1_AR, suppressing its traffic to lysosomes. Besides, negative influences on the cardiac development and overall baseline cardiac performance of mice were not observed when that were lack of USP20. Mice suffering chronic catecholamine stress and pressure overload that are deleterious to heart functions shown higher phosphorylation of USP20 whereas its effects in heart failure still remains unknown [100].

Moreover, carvedilol which can be used to treat chronic heart failure blocks the recycling and the binding of β_2_ARs to USP20, facilitating β_2_ARs’ endocytosis and down-regulation [139]. Furthermore, study has shown that with chronic pressure caused by transverse aortic constriction (TAC), the mice with USP20 knockdown displayed severely deteriorated systolic function in comparison with wide type mice. And augmentation of cardiomyocyte apoptosis, interstitial fibrosis and mouse mortality was observed in mice being lack of USP20. Therefore, USP20 related signaling contributes to the suppression of maladaptive remodeling and prevention of cardiac failure under chronic pressure [140].

Furthermore, Forkhead box P1 (Foxp1) modulates embryonic cardiomyocyte proliferation and heart development. Researchers demonstrated that USP20 which can deubiquitinate HIF1ɑ is a Foxp1 direct target gene and Foxp1 is enriched in promoter regions of USP20. Cardiomyocyte‐specific loss of Foxp1 promotes the USP20‐HIF1α‐Hand1 signaling pathway, promoting a metabolic shift from fatty acid oxidation to glycolysis and ultimately enhancing cardiomyocyte proliferation and heart regeneration, providing novel molecular strategies that can promote heart regeneration and repair for therapeutic intervention in heart failure [101].

Besides heart failure, aging makes a big difference to the integrity of the neurovascular unit (NVU), and it strengthens the sensitivity of NVU to stroke and neurodegenerative diseases [141]. Ischemic stroke is the third leading cause of long-term disability all over the world, and its first-line treatment includes recanalization by intravenous (i.v.) thrombolysis and thrombectomy. However, due to the low ratio of the patients that are eligible for the first-line treatment, further investigations for the complicated pathogenic mechanisms are significant to have better therapy options [142].

The direct interaction between USP20 and phosphatase and tensin homolog deleted on chromosome 10 (PTEN) brings about the lessening of inflammatory signaling concerning NF-κB that is enhanced by middle cerebral artery occlusion (MCAO). The mouse model with MCAO-induced cerebral ischemic stroke shown a decreased level of USP20, while LV-USP20 injection alleviated neuronal death induced by MCAO. Furthermore, in MCAO-treated mice and in OGD/R-stimulated cortical neurons, overexpression of USP20 can mitigate neuronal death through decreasing Caspase-3 activation along with increasing Bcl-2 expression levels. Besides the attenuation of the cerebral ischemic strokes, the cognitive impairments are also demonstrated to be ameliorated by USP20 [103].

Additionally, USP20 can deubiquitinate RIPK1 which is the downstream of TNFR1 and through this process it recedes atherosclerosis by suppressing the activation of NF-κB induced by TNF. NF-κB possesses the capacity of modulating vascular cell adhesion molecule 1 (VCAM1), and adhesion molecules, containing VCAM1, can promote the formation of atherosclerosis in the initial stage. Besides, USP20 can suppress the inflammation of SMCs mediated by atherosclerosis cytokine IL-1β and TLR4. In conclusion, the atherogenic signaling can be reduced by USP20 [104].

Furthermore, for cardiovascular disease such as heart failure, sudden death, and arrhythmia, cardiac hypertrophy is a significant risk factor. The expression of USP20 was observed attenuated in human hypertrophic myocardium of patients with heart failure and ardiomyocyte‐specific knockout of USP20 can aggravate cardiac hypertrophy and dysfunction induced by Ang III. The results direct researchers’ further investigation into the underlying mechanisms and it was shown that through the active site H645, USP20 can attenuate the K63‐linked deubiquitination of STAT3 at residue K177. And then USP20 inhibits STAT3 transcriptional activity in nuclear, contributing to promotion of *Carm1* expression in cardiomyocytes. CARM1 is an essential factor for cardiac homeostasis and can regulate various aspects of cardiomyocyte maturation. Thus, the cardiomyocyte‐specific USP20‐STAT3‐CARM1 axis plays a protective role in cardiac hypertrophy [102, 143].

Furthermore, through interaction with NLRP3 with the USP domain of USP20, USP20 can deubiquitinate NLRP3 by removing K63‐linked ubiquitin at the K243 residue via its active site C154, contributing to suppression of NLRP3 activation and subsequent pyroptosis. USP20 then ameliorates septic myocardial injury and dysfunction. Most mouse models of septic cardiomyopathy are established induced by LPS, and we have observed a significant reduction of both mRNA and protein levels of USP20 in the hearts of mice induced by LPS. And in myocardial tissue with overexpression of USP20 caused by AAV9 virus carrying USP20 cDNA, we can see that LPS induced elevation in serum levels of cTnI, CK‐MB, and LDH can be reduced, indicating potential opportunities of USP20 in treating septic cardiomyopathy [105].

Moreover, in the hearts of mice subjected to aortic banding‐induced cardiac hypertrophy, USP10 expression is prominently increased. Study shown that USP10 physically binds to sirtuin 6 (Sirt6) and Sirt6 mediates the effect of USP10 on the regulation of cardiac hypertrophy and deficiency of USP10 leads to the aggravation of cardiac hypertrophy [144]. And investigators shown through downregulating USP10, acacetin significantly ameliorated cardiac remodeling, indicating a promising candidate drug for the treatment of cardiac remodeling induced by pressure overload [106]. The experiment we discussed above shown the influences of DUBs in cardiovascular diseases.

### Roles of DUBs in metabolic diseases

Aging has something to do with the elevation of total cholesterol (TC) and LDL-cholesterol, and it’s relevant with the decrease of bile acid synthesis, which can lead to hypercholesterolaemia. Moreover, TC and LDL-cholesterol are positively related to all-cause mortality [145]. Additionally, what has been well-documented is that the elevated level of plasma cholesterol can play a vital role in the occurrence of cardiovascular diseases (CVDs), liver disorders such as non-alcoholic fatty liver disease (NAFLD), non-alcoholic steatosis hepatitis (NASH), and metabolic diseases [146]. The synthesis process of cholesterol consists of a series of ~ 30 reactions and during these procedures, hydroxy-3-methylglutaryl-CoA reductase (HMGCR) functions as one of the pivotal rate-limiting enzymes [147].

Study shown that being injected with glucose and insulin after the wild-type mice were fasted resulted in the increase of HMGCR protein level while this was not observed in the hepatocytes that were USP20-deficient. Meanwhile, mTORC1 inhibitor made contributions to the inhibition of the accumulation of HMGCR which was mediated by the deubiquitination of USP20. Further investigation illustrates that mTORC1 activated by refeeding can phosphorylate USP20 at S132 and S134, and then the phosphorylated USP20 makes a connection with gp78 and leads to the deubiquitination and stabilization of HMGCR, which will bring about the elevation of cholesterol. Treating wild-type mice with GSK2643943A which is a specific USP20 inhibitor does not influence the food consumption and nutrient absorption but it can give rise to the reduction of body weight and increase of succinate and then subsequently promote thermogenesis [107, 148]. Silencing of USP20 caused by *si-USP20* effectively reduces the level of HMGCR and body weight without altering food intake in mice fed with high-fat diet (HFD). Moreover, knockdown of USP20 displays a lower lipid level without influencing renal and liver functions [149]. In conclusion, USP20 contributes to the accumulation of HMGCR which then leads to the elevation of cholesterol, and the process uncovers that USP20 can take significant part in the metabolic diseases.

Additionally, as a global health concern, obesity causes abnormal lipid metabolism which can lead to hyperlipidemia. The sleeve gastrectomy (SG) is the most frequently performed bariatric surgery. Study has shown that SG is followed by reduction of both the mRNA and protein levels of USP20, contributing to the attenuation of lipid accumulation, and the consequence is mediated by the decreased deubiquitination of HSPA2 caused by USP20 reduction [109]. Thus, targeting USP20 may be a potential therapeutic approach in treating obesity and lipid accumulation.

As a chronic skeletal disease, osteoporosis is the major cause of fractures in aging people, in which bone resorption and bone formation are imbalanced. Protein disulfide isomerase family A, member 3 (PDIA3) has the ability to enhance osteogenic differentiation of preosteoblast MC3T3-E1, during which, the demethylase the fat mass and obesity-associated protein (FTO) can suppress PDIA3 mRNA methylation, inducing the stabilization of PDIA3 mRNA stability. In addition, silence of USP20 contributes to reduction of FTO, which can be reversed by proteasome inhibitor MG132, illustrating that USP20 can improve the level of FTO. Furthermore, through activating PKA, PDIA3 enhances PKA-mediated phosphorylation, an essential process for USP20 activation. In conclusion, USP20, FTO and PDIA3 constitute a positive feedback regulatory loop, functioning significantly on osteogenic differentiation and offering a potential novel target for treating osteoporosis [108].

Besides USP20’s functions in metabolic diseases, USP19 has been demonstrated modulating adipogenesis and potentiating high-fat-diet-induced obesity and glucose intolerance in mice and USP19 depletion results in body weight reduction [110]. Similar to USP20, USP2, 7, 15 and 19 stimulate either adipogenesis or lipid synthesis, contributing to obesity. While in contrast to USP20, researchers shown that individuals with high USP53 expression in adipose tissue had a markedly decreased body mass index during the dietary intervention indicating that USP53 might be restorative for obesity [111].

### Roles of DUBs in immune response

Adaptive and innate immune response have been observed declined with ageing, which is also related with chronic inflammation status and increasing possibility of autoimmune diseases [150]. Containing Toll‐like receptors (TLRs), retinoic acid‐inducible gene I (RIG‐I)‐like receptors (RLRs), and DNA sensor cyclic GMP‐AMP (cGAMP) synthase (cGAS), pattern recognition receptors (PRRs) can recognize pathogen‐associated molecular patterns (PAMPs), contributing to the activation of innate immunity. The wide participation of ubiquitination in the innate immune signaling cascades has been demonstrated [9].

The gene depletion studies illustrated that the nucleotidyl transferase cyclic AMP-GMP synthase (cGAS) plays a vital role in defensing DNA viruses in mice and various types of cells. The synthesis of cyclic dinucleotide (cGAMP) of 2’-5’-linkage phosphodiester can be accelerated by cGAS mediated by its connection to DNA. And sequentially, cGAS binds to ER and mitochondrial adaptor protein stimulator of interferon gene (STING), which induces the recruitment of adaptor proteins including tumor necrosis factor receptor-associated factor 6 (TRAF6) and kinases TANK-binding kinase 1/IκB kinase ε (TBK1/IKKε) and IKKα/β/γ complex and the transcription factors’ activation eventually. The molecules discussed above possess the capacity of inducing the downstream genes involving IFNs expression [112].

MITA, also called stimulator of interferon gene (STING), was observed to interact with USP18 initially and the DUB activation of USP18 was dispensable during the antiviral signaling. Further study demonstrated that USP18 recruited USP20 to function on STING, during which USP20 interacted with the C-terminus (aa 151-372) of USP18 while STING interacted with the N-terminus (aa 1-150) of it. Additionally, both of USP18 and USP20 can bind to STING N-terminus (aa 1-160). Through deubiquitinating STING by removing the K-48 linked ubiquitin chains from STING, USP20 takes positive part in antiviral response triggered by DNA viruses such as HSV-1, while for RNA-virus triggered signaling USP20 is not indispensable. Furthermore, in mice depleted of USP20, HSV-1 infection is more susceptible to occur [112, 151]. In addition, USP20 can suppress the viral pathogenesis caused by human T cell leukemia virus type 1 (HTLV-1) via its effects on Tax and TRAF6 [90].

With agonist induction, Toll-like receptor 4 (TLR4) and IL-1R dimerize, contributing to the activation of the E3 ubiquitin ligase TRAF6 including oligomerization and autoubiquitination. Lys-63-linked polyubiquitin chains of the activated TRAF6 leads to the activation of TGF-β-activated kinase 1 (TAK1) and IKKβ subsequently. Eventually, IKKβ phosphorylates and mediates the degradation of IκBα, giving rise to the activation of NF-κB signaling [152]. For instance, ubiquitination of adaptor TRAF3 with Lys33‐linked Ub‐chain contributes to ensured temporal and spatial accuracy in response to innate immune signaling and Met1‐/Lys63‐linked ubiquitination induces NF‐κB essential modulator (NEMO) compartmentalization to effectively activate NF‐κB signaling [153, 154].

β-Arrestin2 can inhibit NF-κB activation by impeding the activation of TRAF6 or suppressing IκBα degradation. USP20 can facilitate the deubiquitination of TRAF6 and β-arrestin2, and acting as an adaptor, non-ubiquitinated β-arrestin2 is indispensable for mediating the association between USP20 and TRAF6. In smooth muscle cells (SMCs), USP20 can attenuate TLR4 induced NF-κB activation and USP20 inhibition brings about the enhancement of vascular inflammation induced neointimal hyperplasia [113]. Additionally, phosphorylation of USP20 at S334, which can be induced by IL-1R-associated kinase1 (IRAK1), results in the decreased interaction between USP20 and TRAF6 and enhanced NFκB signaling activation, and ultimately promotes arterial inflammation and neointimal hyperplasia [155]. In conclusion, USP20 participates in the immune response of the body, and through its function of deubiquitination, it can affect the antiviral response and the inflammation closely associated NF-κB signaling.

Furthermore, the recruitment of Tumor necrosis factor receptor 1 associated death domain protein (TRADD) can be improved by the ligation of TNFR, contributing to the interaction between E3 ubiquitin ligases cIAP1/2, TRAF2/5 with the protein kinase receptor-interacting protein kinase 1 (RIPK1). And these molecules are included in Complex Ⅰ. Then, ubiquitinated RIPK1 will be recruited to NEMO, which accelerates the formation of the complex composed of TAK1 and IKK. The process following is the same as IL-1β and TLR4 induced NF-κB signaling activation as we have discussed above. And among the related molecules, RIPK1 can also be deubiquitinated by USP20 [104, 156].

Atypical protein kinase Cs (aPKCs) play a positive role in the modulation of IKKβ activity. The results in the previous investigations shown that during the activation process induced by TNF-α, p62 links with TRADD and this is mediated by RIPK1, and p62 can then act as the bridge linking the aPKCs to RIPK and aPKCs promote the linking of IKKβ to p62 [157]. As we have introduced above, USP20 can mediate the deubiquitination and stability of p62, which is referred to be as the scaffold molecule in PKCζ-mediated NF-κB activation. In the presence of TNF-α, the reduction of IKKα/β phosphorylation and the delay of IκBα degradation are observed when there is lack of USP20 or p62. Additionally, with the depletion of USP20, the expression of NF-κB-mediated target genes like *BFL1* and *cFLIP* decrease. Taken together, during TNF-mediated NF-κB activation, USP20 functions as the positive regulator in the stabilization of p62 [114].

Furthermore, the USP20-p62 axis functions on the RIPK1 upstream, and depletion of USP20 under the treatment of TNFα can lead to the release of RIPK1 from Complex Ⅰ. Then, RIPK1 promotes the formation of Complex Ⅱa and Complex Ⅱb, which results in the cell apoptosis and blocks the cell survival mediated by NF-κB activation [114, 158].

Besides, researchers have demonstrated the inhibition of TRAF3/6 caused by OTUB1/2 and the activation of the same molecule induced by USP25. Furthermore, similar to USP20 which can deubiquitinate STING, the stabilization of STING can also be induced by USP44 and CYLD, indicating the DUBs’ influences on immune response [9]. Furthermore, chronic inflammatory and autoimmune diseases are generally characterized by a prolonged and persistent pro‐inflammatory state and USP4 can promote the stability of nuclear receptor retinoid‐elated orphan receptor‐γt (RORγt) in activated Th17 cells, then induce the production of IL‐17A, one of the pro‐inflammatory cytokines. And in rheumatoid arthritis patients, we can see increased expression of USP4, IL‐17A, and IL‐17F in CD4^+^ T cells [115]. All above indicate the critical functions of DUBs in immune response of elderly patients.

### Roles of DUBs in autophagy

Autophagy incorporates constitutive autophagy which plays a significant role in removing damaged and senescent organelles and maintaining basal energy balance and adaptive autophagy that can mobilize intracellular nutrients when deficient of nutrient. That is to say, autophagy contributes to the energy balance in metabolic process [159].

Autophagy-related (ATG) genes mediate the lysosomal degradation pathway of autophagy, based on which, cellular, tissue and organismal homeostasis are modulated [160]. Possessing serine/threonine kinase activity, uncoordinated 51-like kinase 1 (ULK1) shares homology along its entire length with ATG1 [161]. In autophagy, ULK1-Atg13-FIP200 complex takes essential part, and mTORC1 can mediate the phosphorylation of ULK1 and Atg13 which will suppress the autophagy when there exists enough nutrient [162].

Upon starvation, ULK1 protein dissociates from mTORC1 and the inhibitory phosphorylation of ULK1 is decreased, contributing to itself and Atg13 phosphorylation during autophagy induction. The depletion of USP20 which mediates the deubiquitination of ULK1 leads to the intrinsic instability of ULK1, then reduced phosphorylation of ULK1 and Atg13 occurs without USP20, indicating that USP20 functions positively in the autophagy process. Furthermore, a later time after the induction of autophagy, USP20 dissociates with ULK1, which may be meaningful for the transition to the next steps of autophagy. Through participating in the autophagic process positively, USP20 regulates the cell death induced by starvation, while lack of USP20 results in the increase of apoptotic cell death [116]. Another investigation revealed that the ubiquitin ligase HERC2 can associate with USP20. And p38 can improve USP20 phosphorylation, which leads to the dissociation of HERC2-USP20 interaction and consequently elevates USP20 level. Ultimately, ULK1 deubiquitination and stabilization are achieved due to USP20 and autophagy initiation is promoted [163].

Reticulophagy/ER-phagy, functioning on maintaining ER homeostasis, is a process during which certain ER fractions are selectively degraded. RETREG1 is one of the reticulophagy receptors, whose activation is indispensable in macro-reticulophagy. Moreover, RETREG1 contains a LC3-interacting region which can link to Atg8 family proteins. Study has shown that USP20 can deubiquitinate RETREG1 and strengthen the binding of RETREG1 to LC3B, which consequently promotes reticulophagy especially under starvation condition. Moreover, VAPs, which can facilitate ER-phagophore contact through binding with WIPI2, lead to the ER localization of USP20 mediated by its interaction with FFAT motif of USP20. Although there is lack of the USP20 FFAT motifs, the deubiquitination of RETREG1 caused by USP20 is still observed. The absence of ER localization of USP20 results in the reduction of binding of RETREG1 to LC3B. And with USP20 overexpression, not only the interaction between RETREG1 and VAPB, but also the colocalization of RETREG1 with the early autophagy protein WIPI2 is enhanced, indicating that through enhancing the interaction between RETREG1 and VAPB, USP20 promotes reticulophagy by recruiting early autophagy proteins including WIPI2 [117].

Besides, phosphorylation of PRKN/parkin’s ubiquitin and ubiquitin-like domain caused by PINK1 promotes the activation of PRKN/parkin, being essential for inducing mitophagy which contributes to the damaged mitochondria elimination. USP33 can remove K6, K11, K48 and K63-linked ubiquitin chains from PRKN, taking negative part in PRKN’s functions on mitophagy. Contrarily, silence of USP33 functions positively on both PRKN protein stabilization and its translocation to depolarized mitochondria, leading to mitophagy enhancement [118]. Different form the functions of USP33 in mitophagy, USP10 can promote ULK1 transcription by interacting with and stabilizing GSK3β through deubiquitination, and then increase the activity of the ULK1 promoter, contributing the promotion of autophagy [119]. All above indicates the influences of DUBs in autophagy.

## Therapeutic targeting of the ubiquitin system in ageing-related diseases

In accordance with the functions of ubiquitination on ageing-related diseases we have discussed above, small shifts in either ubiquitination enzymes or deubiquitinating enzymes contribute to significant differences in relevant substrates and relative ageing-related disorders. Here, we concentrate on the therapeutic targeting of the ubiquitin system in ageing-related diseases. We will introduce the regulation mechanisms of DUBs firstly, and then give brief illustration of PROTACs, modulation of mitophagy and autophagy, drug repurposing which are closely associated with ubiquitination sequentially.

### Mechanisms of modulating E3 ligases and DUBs

#### Mechanisms of modulating E3 ligases

As we have discussed above, E3 ligases are divided into two major families including the HECT E3s and the Cullin RING E3s (CRLs). Here we will introduce the modulation of CRLs at the beginning. Under physiological conditions, CRLs’ association with NEDD8, Cullin-associated NEDD8-dissociated protein 1 (CAND1) and CSN are indispensable for CRLs’ functions. In the process of NEDDylation, covalent conjugation between NEDD8 and a specific conserved lysine residue at the Cullin CTD can be observed [164]. Not merely CRL activity and the ubiquitination of substrates but also the association between E2-Ub conjugates and CRL can be modulated by NEDDylation [165, 166]. And the potential mechanism has been illustrated that the modified Cullin subdomain can be reoriented and the structural flexibility of the Rbx1 RING domain can be changed significantly through Cullin NEDDylation [167]. And NEDDylation is a reversible process which can be revered by CSN complex [168].

Also reversibly, CAND1 can bind to unNEDDylated Cullin scaffold and modulate the CRL assembly through competing with the adaptor subunit [169–171]. And investigators proposed that the substrate binding can stimulate NEDDylation while block CAND1 association, contributing to the stabilization of CRL. However, the functions of the binding can be reversed by the ubiquitination and targeting for degradation of the substrates [172]. Furthermore, oligomerization of the E3 complex is required for CRL functioning and activation in some cases, which has been demonstrated having the capacity of regulating activation and enhancing substrate ubiquitination [172].

Involved mechanisms that can regulate HECT E3 ligases’ functions will be illustrated in four perspectives. Firstly, adaptor proteins induced recruitment of HECT E3s to their substrates can make a difference to the substrate-specific conjugation of ubiquitin. Take one of the HECT adaptors --- viral protein E6 as a classic example. The association between viral protein E6 and E6AP can not only influence the substrate recognition but also augment the catalytic activity of the HECT E3 [173]. Besides, HECT E3s catalytic activity can be modulated by E2-conjugating protein recruiting. For instance, SMAD7 can recruit the E2-conjugating enzyme of SMURF2 UbcH7, contributing to the improvement of the ubiquitin ligase activity of E3 [174].

Then we will give brief introductions of inter‑ and intramolecular interactions, which are helpful for HECT E3 ligases’ catalytic activity regulation. For instance, the N-terminal domain and the HECT domain of NEDD4 E3 ligases can interact with each other, contributing to the inactive conformations of the HECT E3 ligases themselves [175]. Moreover, to modulate HECT E3s, cells can attenuate auto-inhibitory interactions and increased Ca^2+^ concentration which can disrupt the auto-inhibitory conformations of NEDD4.2 is an instance [176]. Additionally, oligomerization and E6AP trimerization play a positive role in the ligase activity of HECT E3s [177].

Moreover, through altering conformation or the interactions between HECT E3s and adaptors or other regulatory proteins, post-translational modifications can make a difference to activation of HECT E3s. Phosphorylation can influence the activation of HECT E3s both in positive and negative ways. For instance, phosphorylation of a PRR of ITCH can relieve ITCH from its auto-inhibitory fold, activating the HECT domain [178]. While contrary effects can be induced by phosphorylation on E6AP [179]. Besides, ubiquitination [180], SUMOylation [181], NEDD8 modification [182] and ISG15 [183] can also influence the activation of HECT E3s with different mechanisms.

#### Mechanisms of modulating DUBs

Transcription, translation and degradation can modulate the abundance of DUBs, whose expression can be induced relying on relevant stimulation. For instance, it has been shown that A20 is a tumor necrosis factor (TNF)-induced gene and expression of A20 can be regulated by mucosa-associated lymphoid tissue lymphoma translocation protein 1 (MALT1). Also modulated by MALT1, CYLD is a NF-κB-regulating DUB, which can be targeted by capase-8 [184–186].

Besides of DUB abundance, subcellular localization is another factor regulating DUB functions, and various of mechanism such as targeting domains to interact with distinct cellular membranes can help to achieve DUB localization. Furthermore, kinase induced phosphorylation results in diverse consequences of DUB localization. For instance, ATM induced USP20 phosphorylation brings about its nuclear localization while Akt-mediated USP4 phosphorylation contributes to its exclusion from nuclear. Additionally, localization of DUBs can also be influenced by alterations of DUB engaging protein interactions [187–189].

**Fig. 4:**
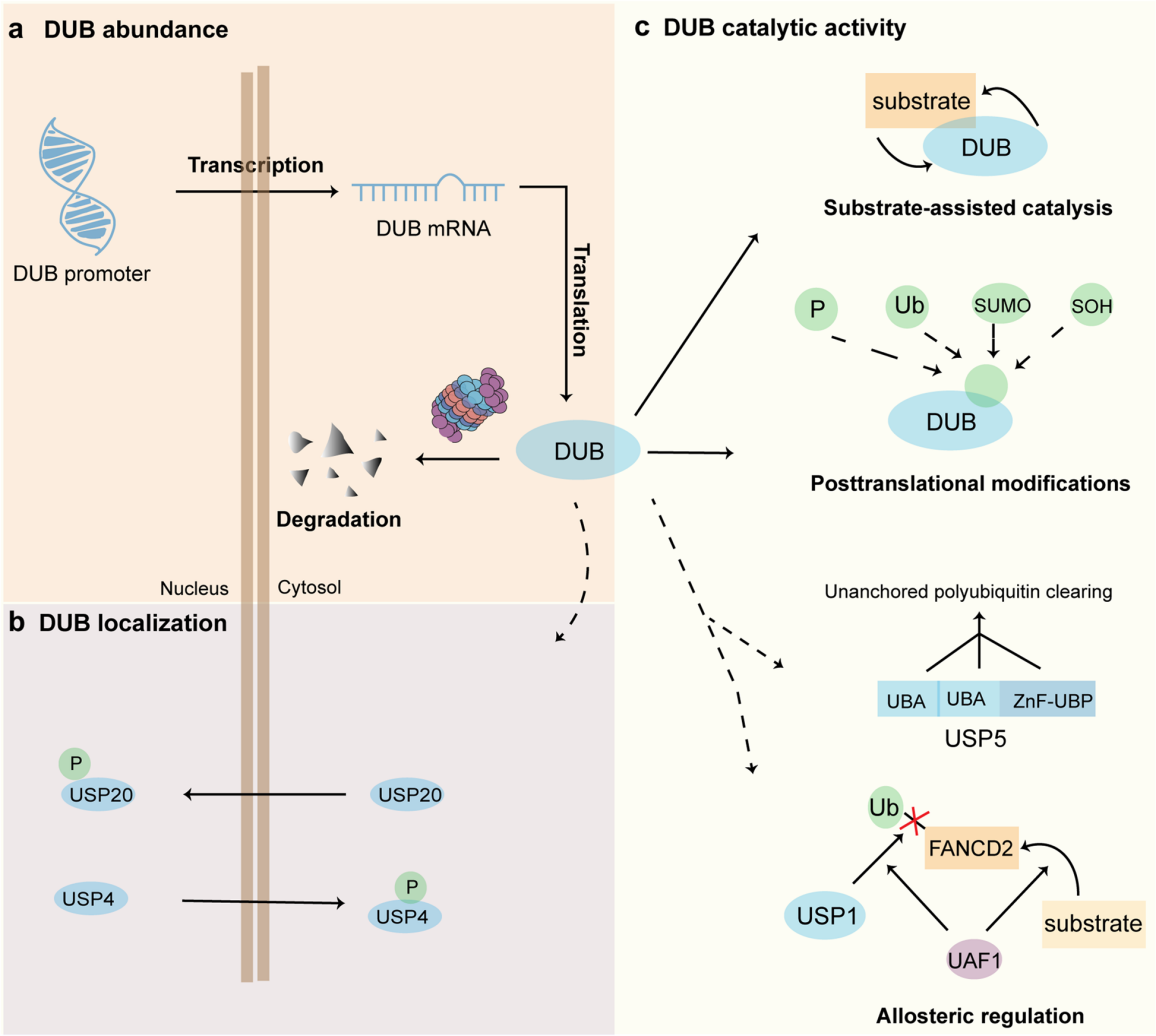
Mechanisms of regulating DUBs. Functions of DUBs can be regulated through modulating DUBs’ abundance, localization and catalytic activity. **a** Abundance of DUBs can be regulated by transcription, translation and degradation. **b** DUB localization can be influenced by varieties of means including posttranslational modifications and protein interactions. For instance, ATM induced USP20 phosphorylation brings about its nuclear localization while Akt-mediated USP4 phosphorylation contributes to its exclusion from nuclear. **c** Substrate-assisted catalysis, allosteric regulation and posttranslational modifications involving phosphorylation, ubiquitination, SUMOylation and oxidation play a significant role in regulation of catalytic activity of DUBs. For allosteric regulation, two ubiquitin-associated (UBA) domains and a C-terminal ZnF-UBP domain enable specific interactions with unattached ubiquitin chains, promoting proximal exo-cleavage. Moreover, USP1 can be activated by UAF1 and USP1 can target ubiquitinated FANCD2. UAF1 not only has the capacity of enhancing USP1 catalytic activity but also can recruit FANCD2 substrates

As for DUB catalytic activity, substrate-assisted catalysis, posttranslational modifications and allosteric fashion that can make a big difference are introduced in sequence (Fig. 4). To begin with, disability of OTULIN to cleave Lys63-linked chains can be seen despite the enzyme concentrations, while OTULIN can be activated by Met1 diubiquitin shown by its crystal structures [190, 191].

Further illustration concerning influences of posttranslational modifications involving phosphorylation, ubiquitination, SUMOylation and oxidation on DUBs is given. Activation of CYLD can be modulated by phosphorylation positively or negatively, and can also enhanced by IKKβ phosphorylation [192–194]. In addition, DUBs can be ubiquitinated mediated by at least one but often multiple ubiquitin-binding sites and UCH-L1 is a classic example. At lysine residues around the active site, UCH-L1 can be monoubiquitinated, inducing its catalytic activity alteration [195, 196]. Some USPs including USP25 and USP28 harbor a SUMO-interacting motif in which Lys99 and Lys141 can be SUMOylated, impairing ubiquitin binding and cleavage [197, 198]. Moreover, oxidation caused by reactive oxygen species can be seen in inactive members of the OTU, USP, and UCH families in vitro and vivo [199–201].

Allosteric modulation will be introduced from three aspects involving the effects of domains within the enzyme, the DUB interactome and macromolecular complexes. To begin with, we take domains of USP5 as an instance. The C-terminal ZnF-UBP domain of USP5 takes positive part in influencing unanchored polyubiquitin clearance from cells while the N-terminal of the domain can enhance its isopeptidase activity. And removal of the N-terminal domain in USP5 from its catalytic USP core contributes to activity reduction toward ubiquitin-AMC [202–204]. As for DUB interactome, USP1 can be allosterically activated by UAF1 and target ubiquitinated FANCD2 and PCNA. Moreover, UAF1 not only has the capacity of enhancing USP1 catalytic activity but also can recruit FANCD2 and PCNA substrates, indicating that the DUBs act as part of protein complexes [205–208].

We take SAGA complex, being responsible for histone deubiquitination, as an instance for macromolecular complex. It has been demonstrated that the incorporation with SAGA DUB module is indispensable for USP22 activation. Furthermore, the association between the crystal structure of the SAGA DUB module and a monoubiquitinated nucleosome provides molecular basis for substrate recognition, indicating that the substrate is contacted by both the SAGA component and USP domain itself [209–211].

### Small-molecule inhibitors and PROTACs targeting ubiquitin machinery

We have discussed diverse mechanisms through which E3 ligases and DUBs can be regulated, suggesting that E3 ligases and DUBs can be targeted selectively. Here we will give brief introductions of small-molecule inhibitor of DUBs, which can be classified into five types. Type Ⅰ DUB inhibitors are those can interact directly with catalytic-site residues and stabilize the target DUB in an active conformation, with the active site residues aligned for catalysis, such as small molecules constitutive photomorphogenic-9 signalosome subunit 5 (CSN5) that target the metalloprotease DUB [212]. In contrast with type Ⅰ DUB inhibitors, type Ⅱ DUB inhibitors interact directly with catalytic-site residues while stabilize the target DUB in an inactive conformation, with misaligned active-site residues. For instance, the small molecule FT827 can form a covalent bond with the catalytic cysteine of USP7 and it is capable of stabilizing USP7 in a catalytically misaligned conformation [213].

Type III DUB inhibitors are competitive molecules that bind to the Ub-binding sites of DUBs and block substrate recognition but do not interact directly with catalytic residues, among which Type III-D that can target the S1-binding pocket and block the distal Ub from binding are the largest class of DUB inhibitors. Furthermore, Type III-D possesses the most potent and specific binders reported to date and the first reported crystal structure of this type was the small molecule GRL0617 in complex with the PLpro domain of SARS-CoV [214]. The type IV DUB inhibitors that are allosteric inhibitors outside of Ub-binding sites and type V DUB inhibitors to be bivalent molecules that simultaneously bind two regions of a DUB have not been identified, while it is attractive strategy to developing type IV and type V inhibitors for DUBs [215].

A meaningful technology used for target protein degradation named proteolysis-targeting chimera (PROTAC) is introduced then. Composed with both of ligands for protein of interest and an E3 ubiquitin ligase, a bifunctional PROTAC molecule can recruit E3 ligases after binding to protein of interest, inducing the proteasome-mediated degradation. Despite the existence of a great number of E3 ligases, only the ligases with small molecule ligands have been used for designing PROTACs involving VHL1, MDM2 and so on. The first PROTAC ternary complex RBD4-MZ1-VHL strongly and selectively targets RBD4 and results in the degradation of RBD4. Moreover, in several leukemia cells, researches shown potential anti-proliferation activity of RBD4-MZ1-VHL [216, 217].

Besides RBD4, many other proteins of interest are devoted to design PROTACs including Bruton’s tyrosine kinase (BTK), which associates with survival and proliferation of B-cell neoplasms, BCR-ABL, whose activity leads to the oncogenesis of chronical myeloid leukemia, and MCL1, whose overexpression has been observed in lymphoma, leukemia and MM. Furthermore, PROTAC probes involving ARV-110 and ARV-471 have been in clinical trials for prostate and breast cancer respectively [217].

Moreover, deubiquitinase Targeting Chimera (DUBTAC), developed from a powerful therapeutic modality---targeted protein degradation, can stabilize the levels of specific proteins degraded in a ubiquitin-dependent manner and then influence the progression of relevant diseases [218]. Nowadays, the first USP7-based DUBTACs has been developed and it has been demonstrated that the DUBTACs can contribute to the stabilization of AMPKβ1 and then affect tumor cell growth [219].

### Enhancing autophagy for anti-ageing intervention

Just as we have discussed above, there exist close relationship between ubiquitin proteasome system, DUBs and mitophagy and autophagy. Here we will give further investigations of whether autophagy can be enhanced through regulation of ubiquitin proteasome system and DUBs and then result in anti-ageing intervention.

Autophagy functions differently in diverse phases of cancer progression. In the onset and early stages of tumor development, autophagy impairment contributes to cancer progression. While in the later stages, cancer cells can use autophagy to cope with the intracellular stress associated with their malignant state and difficult microenvironmental conditions, promoting cancer metastasis [220, 221]. As for the association of ubiquitination and autophagy in cancers, we take ULK1 as an example. ULK1 not only can be degraded through ubiquitination in normal conditions, but also regulated by ubiquitination in cancers. Previous investigations shown that NEDD4L can decrease cellular ULK1 and ASCT2, and then contribute to the inhibition of autophagy and mitochondrial metabolism, resulting in the inhibition of pancreatic cancer cells’ proliferation and viability [222].

In cancer treatment, several drugs have been discovered to target E3 ubiquitin ligases to modulate autophagy for cancer therapy and targeting the proteasome have already been applied in clinical practice. And with the researches continuing and drug resistance emerging, the advantages of combination therapies are gradually demonstrated. In esophageal squamous cell carcinoma tumorigenesis, PLCE1 can activate MDM2-dependent ubiquitination and degradation of p53, with decreasing autophagy observed [223]. And autophagy exerts anti-tumor effects along this pathway. Additionally, as one of the MDM2 inhibitors, MI-63 possesses the ability to boost the effectiveness of bortezomib and lenalidomide and induce autophagy-linked apoptosis in both p53 wild-type and mutant models of MM [224]. Accordingly, MI-63 may provide insight into ESCC and MM therapy.

Moreover, proteolytic pathway is a promising target for destroying cancer cells and Bortezomib has been proven to be a clinical anti-cancer drug [225, 226]. The Fms-like tyrosine kinase-3 receptor (FLT3) internal tandem duplication (ITD) is associated with a poor outcome of AML and it can be observed in autophagosome in AML cells treated with bortezomib, indicating that the early degradation of FLT3-ITD depends on autophagy induction and then cell death is induced [227]. Furthermore, in pancreatic and colorectal cancer cells, a protective autophagy response can be induced by Bortezomib mediated by AMPK-ULK1 signaling activation [228]. All investigations above indicate the proteasome inhibitors’ potential therapeutic functions in cancers related autophagy.

Furthermore, combination of autophagy enhancer and proteasome inhibitors is increasingly focused on by researchers. Being isolated from a Chinese herb Solanum nigrum L., Solamargine (SM) exhibits promising anti-multiple myeloma (MM) activity. In MM cell lines, genes associated with cell death and autophagy were upregulated after treatment with SM. Besides, autophagy inhibitor contributed to alleviation of SM-triggered apoptosis and inhibition of viability in MM cells, indicating that SM activated autophagy in MM cells. Additionally, in the study, we can observe a synergistic effect between SM and bortezomib in both MM cells and human bone marrow CD138 + primary myeloma cells [229], suggesting the therapeutic potentials of autophagy enhancer and proteasome in MM. The researches we discussed above give us potentials of promoting autophagy through regulation of UPS and DUBs to treat ageing-related diseases.

Besides cancers, therapeutic potentials of neurodegenerative diseases are also in requirement. Neurodegenerative diseases are featured by some proteins’ aggregation, which can be targeted for degeneration by the ubiquitin–proteasome system and the autophagy-lysosomal pathway (ALP) in common cases. Study shown that by upregulating the E3 ubiquitin ligase PELI1 in microglia, fibrillar α-Syn contributed to autophagy impairment, contributing to ubiquitin-mediated degradation of lysosome-associated membrane glycoprotein 2 through UPS and transmission of toxic α-Syn to healthy neurons [230, 231]. Additionally, in Huntington disease models, mTOR suppression can induce autophagy and reduce toxicity of polyglutamine expansions, providing autophagy inducement chances to treat Huntington disease [232]. Thus, whether there exist opportunities of enhancing autophagy through regulating UPS to treat neurodegenerative diseases need further investigations.

### Drug repurposing opportunities targeting UPS

Compared with the time-consuming and costly de novo drug discovery, drug repurposing, referring to identifying existing drugs for new use, is believed to offer great benefits [233]. And for targeting UPS, high-throughput screening (HTS) assays are gradually used as a helpful tool in drug repurposing [234], and we will give several examples of old drugs being new candidate UPS inhibitors.

Aldehyde dehydrogenase is essential during the metabolism of alcohol and the enzyme can be inhibited by Disulfiram (DSF), contributing to DSF being the first FDA approved drug for treating alcohol dependence [235]. Besides, DSF’s potentials to treat various human cancers in vitro and in vivo due to its inhibition of UPS have also been demonstrated [236, 237]. A cell-based screening assay and several studies have illustrated the proteasome inhibition function of DSF especially its strong inhibitory effect on 26S proteasome activity in various cancer cell lines [237]. Moreover, being able to penetrate blood-brain barrier, DSF is concentrated on in clinical trials to evaluate its effectiveness in patients with different stages of glioblastoma (GBM) such as NCT01907165, indicating promising prospects for repurposing this compound as an effective anticancer drug [238].

Besides DSF, several HIV-protease inhibitors involving ritonavir and saquinavir have been demonstrated capable of inhibiting proteasome [239, 240]. Being effective for treating depression, lithium can also suppress GSK3, contributing to the investigation of applying lithium to treat cancers. Furthermore, that lithium chloride can inhibit the CT-L and PGPH-L activities of both the 20S and 26S proteasome specifically can be observed in relevant researches [241, 242]. Thus, further investigations concerning the functions of these old drugs on diverse ageing-related diseases due to their new identified targets are still in requirement. With the advantages including previously well-known pharmacokinetics and pharmacodynamics; fast drug development cycle; economical, efficient clinical translation of basic research; and easy to get clinical approval [238], drug repurposing provides emerging therapeutic potentials for UPS and DUB associated ageing-related diseases.

### Challenges and considerations in therapeutic specificity and delivery

Although there exist quantities of small-molecule inhibitors of DUBs as we have discussed above, majorities of DUB inhibitors reported have shortages including weak inhibitory activity, undesirable chemical features and poor selectivity across DUB enzyme family [243–245]. Recently, several highly specific USP7 inhibitors targeting the MDM2-p53 axis, which functions significantly in tumorigenesis, have been developed. For instance, mediated by the association with 12 Å from the catalytic center, an inhibitor can attenuate USP7 favored binding of the distal ubiquitin of the Lys48-linked substrates [11, 246]. Moreover, although drug repurposing possesses lots of advantages, its limitations include low target-specificity and off-target effects [238]. UPS and DUBs function significantly and complexly in cellular processes and make a big difference in the progression of diverse ageing-related diseases, indicating the critical meaning of further investigation into the highly selective inhibitors to avoid the drugs’ adverse events caused by the poor selectivity.

Besides therapeutic specificity, we can also observe other challenging huddles in the process of drug development, one of which is drug delivery to the target site. Here, we take the drug delivery system for PROTACs as an example, which can transport PROTACs into diseased sites by packing them into a delivery carrier. Firstly, prodrug-based strategies, including addition of a lipophilic pivaloyloxymethyl group into the imide group of the synthesized CDK2/4/6-targeting PROTAC, providing a folate receptor alpha (FOLR1)-targeting delivery strategy for PROTACs, enable graders to overcome challenges such as unfavorable biopharmaceutical or pharmacokinetic performance through chemical modifications with removable groups [247, 248].

Moreover, it has been reported that nanoparticles can enhance the stability and solubility of loaded cargoes, facilitate transportation across biological barriers and prolong circulation times for better efficacy and safety profiles. Nanomedicine has been used to overcome the limitations of a variety of small molecule drugs and nano-sized formulations including lipid-based, polymeric, and inorganic nanoparticles have been engineered and formulated for PROTAC delivery [249–251].

Besides, natural biomacromolecules, among which proteins are most frequently used, have been used as drug delivery platforms due to their biocompatibility [252]. Inspired by antibody–drug conjugates, researchers developed the first antibody-PROTAC conjugate, tethering a BET degrader (GNE-987) to anti-CLL1 or anti-HER2 antibodies [253]. Compared with antibody–drug conjugates, peptide-based carriers have fewer molecular weights and fewer immunogenic concerns. For instance, polyarginine-based cell-penetrating peptide has been used to modify PROTACs targeting AR and ER for breast and prostate cancer treatments respectively [254]. The delivery system discussed above provides us further investigation directions concerning the clinical application of UPS and DUB associated drugs.

## Clinical trials targeting ubiquitin system for treatment of ageing-related diseases

We have discussed the regulation mechanisms of ubiquitin system and potential therapeutic opportunities in patients suffering ageing-related diseases above. Nowadays, several drugs targeting components of ubiquitin proteasome system and DUBs are gradually assessed in clinical trials and here we will give brief introduction of these inhibitors and relative clinical trials (Table 2; Fig. 1).

**Table 2:** Ubiquitin system related inhibitors in clinical trials

Category	Name	Target	Clinical status	Diseases	ClinicalTrials.gov.ID
Ubiquitinating enzyme inhibitors	TAK-243	UAE(E1)	Phase Ⅰ	Advanced solid tumors	NCT06223542
	MLN4924	Nedd8-Activating Enzyme	Phase Ⅰ	Advanced solid tumors ALL Melanoma Nonhematologic Malignancies	NCT03486314 NCT03349281 NCT01011530 NCT00677170
			Phase Ⅱ	MDS HR-MDS, CMML, low-blast AML Advanced Intrahepatic Cholangiocarcinoma Advanced NSCLC Diffuse Large B-Cell Lymphoma	NCT03238248 NCT02610777 NCT04175912 NCT03965689 NCT01415765
	SAR405838	MDM2	Phase Ⅰ	Solid tumors	NCT01985191
	CGM097	MDM2	Phase Ⅰ	Solid tumors	NCT01760525
	DS3032b	MDM2	Phase Ⅰ	Advanced Solid Tumors or Lymphomas Relapsed or Refractory Acute Myeloid Leukemia	NCT01877382 NCT03671564
	NX-1607	Cbl-b(E3)	Phase Ⅰa	Advanced Malignancies	NCT05107674
Proteasome Inhibitors	Bortezomib	Proteasome	Phase Ⅱ	Hormone Refractory Prostate Cancer Graft-versus-host disease Non-Hodgkin's Lymphoma NSCLC Metastatic Kidney Cancer Malignant Mesothelioma Advanced Soft Tissue Sarcoma High-Grade Gliomas Relapsed or Refractory Neuroblastoma Head and neck cancer Prostate Cancer Breast cancer Colorectal cancer Ovarian Epithelial Cancer Atypical Teratoid/​Rhabdoid Tumors Severe Autoimmune Encephalitis Hepatocellular carcinoma	NCT00183937 NCT00408928 NCT00054665 NCT00075751 NCT00025376 NCT00458913 NCT00937495 NCT00108069 NCT02139397 NCT00425750 NCT00183937 NCT00028639 NCT00051987 NCT00023712 NCT06853080 NCT03993262 NCT00077441
			Phase Ⅲ	Multiple Myeloma T-Lymphoblastic Leukemia (T-ALL) and T-Lymphoblastic Lymphoma (T-LLy) AML Diffuse Large B-cell Lymphoma	NCT00200681 NCT02112916 NCT01371981 NCT01324596
	Carfilzomib	Proteasome	Phase Ⅰ	NSCLC T-Cell Lymphoma ALL/AML Advanced Solid Tumors Systemic Light Chain Amyloidosis Hematological Malignancies Non-Hodgkin Lymphoma	NCT06249282 NCT01336920 NCT01137747 NCT02257476 NCT01789242 NCT00150462 NCT02187133
			Phase Ⅱ	SCLC Lymphoma Advanced Neuroendocrine Cancers Renal Cell Carcinoma (RCC) Peripheral T Cell Lymphoma Prostate Cancer Waldenström's Macroglobulinemia	NCT01941316 NCT00531284 NCT02318784 NCT01775930 NCT03141203 NCT02047253 NCT01813227
			Phase Ⅳ	MM	NCT03934684
	Ixazomib	Proteasome	Phase Ⅰ	Advanced Nonhematologic Malignancies Lymphoma Solid Tumors Light Chain Amyloidosis AML ALL	NCT00830869 NCT00893464 NCT02942095 NCT01318902 NCT02582359 NCT02228772
			Phase Ⅱ	Autoimmune Cytopenia Follicular Lymphoma Multiple Myeloma Mantle Cell Lymphoma Cutaneous and Peripheral T-cell Lymphomas Kidney Cancer Waldenstrom's Macroglobulinemia Chronic Graft-versus-Host Disease Plasma Cell Leukemia	NCT03965624 NCT01939899 NCT02168101 NCT03323151 NCT02158975 NCT03587662 NCT02400437 NCT02513498 NCT02547662
			Phase Ⅳ	MM	NCT03416374
	CEP-18770	Proteasome	-	Refractory/ relapsed MM	NCT01348919
			Phase Ⅰ	Solid Tumours or Non-Hodgkin's Lymphomas	NCT00572637
	Oprozomib	Proteasome	Phase Ⅰ	Solid Tumors	NCT01129349
			Phase Ⅱ	Multiple Myeloma	NCT02072863
	Marizomib	Proteasome	Phase Ⅰ	Lymphoma Non-small Cell Lung Cancer, Pancreatic Cancer, Melanoma or Lymphoma Brain Cancer	NCT00396864 NCT00667082 NCT02903069
			Phase Ⅱ	MM	NCT00461045
			Phase Ⅲ	Glioblastoma	NCT03345095
DUB inhibitors	TNG348	USP1	Phase Ⅰ/Ⅱ Terminated	BRCA 1/2 Mutant or Other HRD + Solid Tumors	NCT06065059
	Pimozide	USP11	Phase Ⅰ	Solid tumors Androgen-Independent Prostate Cancer Lymphoma Ph + Acute Lymphoblastic Leukemia Head and Neck Cancers	NCT02043756 NCT00059631 NCT05173545 NCT00940524 NCT04902027
			Phase Ⅱ	Recurrent NHL or Acute Leukemia HIV Associated Large Cell and Immunoblastic Lymphomas Neuromyelitis Optica Spectrum Disorder (NMOSD) Follicular Non-Hodgkin's Lymphoma Chronic Lymphocytic Leukemia Hematologic Cancers Extranodal Natural Killer/​T Cell Lymphoma T-Prolymphocytic Leukemia	NCT01842672 NCT00002003 NCT05551598 NCT00169208 NCT00274963 NCT00015951 NCT05464433 NCT01186640
			Phase Ⅲ	Metastatic Breast Cancer Prostate Cancer	NCT00002544 NCT00004071
			Phase Ⅳ	Recurrent Neuromyelitis Optica AML	NCT00304291 NCT00180167

Here we introduce ubiquitin system-related inhibitors, including ubiquitin ligase inhibitors, proteasome inhibitors and deubiquitinating enzyme inhibitors, and relevant clinical trials. Additionally, the terminated clinical trials are not involved here

Investigators have shown that E3 ligases function both as tumor suppressors and oncogenic proteins and the underlying mechanisms of regulating cellular processes are complex. Here we give some introductions of inhibitors of ubiquitinating enzymes especially E3 ligases in clinical trials. Being able to connect with NAE to create a NEDD8-MLN4924 adduct, MLN4942, also known as Pevonedistat, can block neddylation of all cullin-RING ligases, contributing to the regulation of intracellular protein destruction. And MLN4924 now has been applied in clinical trials for certain solid tumors and hematologic malignances and shown clinical activity for acute myelogenous leukemia (AML) [255, 256]. Furthermore, pevonedistat shown tolerated results not merely in advanced solid tumors [257], but also in children suffering solid or CNS tumors with combination of chemotherapy irinotecan and temozolomide [258]. Also demonstrated in older AML patients being unfit for high-dose induction therapy, combination of PEV and AZA was well tolerated and shown potential benefits [259].

Mouse double minute 2 (MDM2) can associate with p53 and suppress its activity, increasing cell survival in varieties of cancers, while Nutlin-3a can suppress the interaction. Derivatives generated from Nutlin-3a optimization including RO-5503781 and MK-8242 were also investigated in clinical trials for treating AML and advanced solid tumor prostate cancer and liposarcomars respectively, while the investigations were terminated. Possessing similar functions, SAR-405838, CGM097 and DS3032b are in clinical trials for solid tumors either in combination with chemotherapy or not [260–262]. However, among these inhibitors, DS3032b, also known as milademetan, has been demonstrated short-lived tumor reduction in refractory MDM2 amplification, TP-53 wild-type solid tumors [263].

The ability of Avadomide to modulate the activation of cereblon E3 ligase brings about its potent activities on tumor suppression and immunomodulation. And the acceptable safety and favorable pharmacokinetics of Avadomide can be observed in phase 1 study enrolled patients with advanced solid tumors, non-Hodgkin lymphoma and multiple myeloma [264].

Besides of inhibitors of E3 ligases, several proteasome inhibitors have been approved by FDA in treating multiple myeloma (MM). Being a distinctive and reversible inhibitor of the 26S proteasome, bortezomib induces the accumulation of polyubiquitinated proteins. It is not only the first proteasome inhibitor introduced for MM, but also can be applied in treating relapsed mantle cell lymphoma, disuse large-B cell lymphoma and colorectal cancer [265]. And in a phase 2 trial with study population suffering unresectable hepatocellular carcinoma, bortezomib monotherapy shown minimal clinical activity and not excessive toxicity. Moreover, in MM patients with resistance to bortezomib, carfilzomib shown stronger inhibition on chymotrypsin-like activity of the proteasome than bortezomib, resulting in its approval as another treatment plan. While carfilzomib does not dissolve well in water, it cannot be administered orally. Ixazomib, which can inhibit the activity of proteasome through binding to catalytic β-subunits, is also approved as the first orally bioavailable proteasome for MM [266].

Furthermore, we give brief illustrations of mall molecular inhibitors against DUBs which have entered in clinical trials. As a reversible inhibitor of the USP1/UAF1 complex, pimozide can function with collaboration of cisplatin, inducing inhibition of cell-proliferation in NSCLC cells with resistance to cisplatin, and clinical trial concerning pimozide is conducting in patients with amyotrophic lateral sclerosis [267, 268]. Also, in patients with MM being refractory to or relapsed after treatment with at least one immunomodulatory and one proteasome inhibitor, the small molecule DUB inhibitor VLX1570 was assessed while its severe pulmonary toxicity can be seen [269].

## Conclusions and prospects

Just as we have discussed before, crosstalk between diverse posttranslational modifications functions significantly in cellular physiological processes. Nowadays, for ubiquitination, the main investigations concentrating on PTM crosstalk contain the association with phosphorylation and SUMOylation. However, we can also observe association between other posttranslational modifications. For example, ubiquitination of p53 caused by the E3 ligase MDM2 can be inhibited through the acetylation of the same lysines of p53 C-terminal domain by p300. Besides, acetylation can also create an acetyl-degron which can target a substrate for proteasomal degradation [270]. Therefore, to further investigate the posttranslational modifications’ crosstalk is still in requirement to better understand the functions and mechanisms of ubiquitination in ageing.

Moreover, we mostly concentrate on the functions of USP20 in ageing-related diseases here. As is known to us all, the mechanisms of neurodegenerative diseases, whose risk factor contains aging as well, are still obscure, while investigations shown that in several neurodegenerative diseases, protein aggravation is the pathological features. And ubiquitin–proteasome degradation can contribute to the removal of these aggregates [271]. And we have shown above that quantities of DUBs have been demonstrated closely associated with neurodegenerative diseases. Neurodegenerative diseases may be implicated potentially by the neurotoxicity in cerebellar granule neurons induced by Manganese [272, 273]. Study has shown that USP20 can promote the growth of axons while inhibit their branching in cerebellar granule neurons [274]. Whether there exists relationship between USP20 and these ageing-related diseases remains to be demonstrated. Additionally, more investigations concerning associations between ageing-related diseases and other ubiquitin proteasome system components are also future directions.

Besides the preclinical studies, it is also significant to concentrate on translational medicine. In addition to the UPS components’ inhibitors assessed in clinical trials, researchers have gradually developed more inhibitors including E2 inhibitor CDC34A, UBA1 inhibitor PYP41 and USP7 inhibitor thiophene [275–277]. For elderly patients especially with cancers and neurodegenerative diseases, these potential therapeutic regiments are of significance. Moreover, for drugs that have been investigated in clinical trials such as VLX1570, although there exists severe toxicity, researchers should further investigate the possible reasons for the toxicity and potential approaches to deal with the adverse agents [269].

Additionally, during the regulation of ubiquitin proteasome system in ageing-related diseases, PROTAC and DUBTAC are increasingly attract researchers’ concentration just as we have discussed above. PROTACs can have good absorption, distribution, metabolism and elimination properties by choosing drug-like ligands of proteins of interest and E3s followed by medicinal chemistry optimization, indicating that PROTACs may be the potential direction of ageing-related disorders in the future. However, reduction of POIs caused by biological activities of a PROTAC is different with the pharmacological inhibition of POIs and PROTACs’ activity depends on its associated E3, whose level is diverse in different tissues and cell types, indicating that there are significant challenges in developing PROTACs as drugs. Thus, further investigations in PROTACs and DUBTACs are still in need [217].

In conclusion, here we firstly give a brief introduction of the ubiquitin system including the ubiquitinating enzymes, deubiquitinases and the processes of ubiquitination/deubiquitination and then we associate ubiquitin system with cellular ageing and homeostasis, which indicate the interaction between ubiquitin system and ageing. Furthermore, we illustrate the detailed mechanisms of influences on ageing-related diseases caused by deubiquitination, and during this part, we especially concentrate on the deubiquitinase USP20’s functions on ageing-related diseases. Also, we introduce some other DUBs’ influences in ageing-related diseases. Moreover, the regulation of E3 ligases and deubiquitinases and relevant clinical trials investigating relevant inhibitors are illustrated which give researchers more directions and elderly patients more potential therapeutic regiments. Taken together, USP20 and other ubiquitin system components appear to be promising candidates for treating aging-related diseases due to their effects on the progression of aging-related diseases. Future work is still needed before it can be considered as viable therapeutic targets, and our review could prospectively contribute to this progress.

## Data Availability

Not applicable.
